# Optimization of extraction conditions for total flavonoids from *Clerodendrum cyrtophyllum* Turcz. and its antibacterial activity and mechanism against *Staphylococcus aureus*

**DOI:** 10.3389/fmicb.2026.1961127

**Published:** 2026-09-21

**Authors:** Yue Hu, Wenwen Luo, Chengjian Tan, Guoshun Pei

**Affiliations:** School of Ethnic Medicine, Guizhou Minzu University, Guiyang, Guizhou, China

**Keywords:** antibacterial activity, antibacterial mechanism, *Clerodendrum cyrtophyllum* Turcz., component analysis, flavonoids, response surface optimization, *Staphylococcus aureus*

## Abstract

**Introduction:**

*Clerodendrum cyrtophyllum* Turcz., a traditional Chinese medicinal plant, exhibits broad bioactivity. Nevertheless, the flavonoid constituents conferring anti-*Staphylococcus aureus* (*S. aureus*) activity and their mechanistic basis remain to be elucidated.

**Methods:**

Total flavonoid extraction from *Clerodendrum cyrtophyllum* Turcz. (TFC) was first evaluated by single-factor experiments to screen key variables and subsequently optimized by a Box–Behnken design (BBD) within response surface methodology (RSM). followed by purification with D101 macroporous resin. Liquid chromatography–tandem mass spectrometry (LC–MS/MS) characterized the chemical profile. Antibacterial activity against *S. aureus* was assessed via inhibition zones, minimum inhibitory concentration (MIC), minimum bactericidal concentration (MBC), and growth curves. Mechanisms were explored by evaluating cell surface hydrophobicity (CSH), extracellular alkaline phosphatase (AKP), leakage of nucleic acids/proteins, membrane potential, ATP levels, reactive oxygen species (ROS) content, and morphological alterations.

**Results:**

The optimal extraction conditions for TFC were determined as 70% ethanol as the solvent, an extraction time of 102 min, a temperature of 64 °C, and a solid–liquid ratio of 1:25 (g: mL), resulting in a yield of 6.48% ± 0.58. After purification, the purity of TFC was increased to 76%, and preliminary analysis identified 85 flavonoid constituents. TFC exhibited inhibition zone diameters of 13 ± 0.45 mm, 16 ± 0.38 mm, and 20 ± 0.45 mm at concentrations of 50, 100, and 150 mg/mL, respectively, with MIC and MBC values of 0.781 mg/mL and 1.562 mg/mL. Notably, TFC effectively inhibited the growth of *S. aureus* within 24 h. Mechanistic investigations revealed that TFC treatment increased *S. aureus* CSH and decreased the membrane potential, leading to the leakage of intracellular nucleic acids and proteins, indicating damage to *S. aureus* cells, which was further confirmed by SEM. Furthermore, TFC induced significant intracellular ATP depletion, decreased intracellular nucleic acids and protein contents, and triggered excessive ROS production.

**Conclusion:**

This study optimized the extraction conditions of TFC and successfully obtained TFC rich in multiple flavonoid constituents. TFC exerts potent inhibitory effects on *S. aureus* by disrupting the integrity of cell walls and membranes, depleting intracellular energy reserves, decreasing the contents of intracellular nucleic acids and proteins, and inducing intracellular oxidative stress.

## 1 Introduction

*Staphylococcus aureus* (*S. aureus*), a ubiquitous infectious pathogen, represents a significant threat to both public health and livestock production (Cheung et al., 2021). It is capable of causing a wide spectrum of infectious diseases across all age groups, with particularly severe outcomes in immunecompromised individuals. When host immunity is compromised or skin mucosal barriers are breached, *S. aureus* can invade the bloodstream and disseminate to various tissues, potentially leading to systemic infections affecting nearly all organ systems (Kouijzer and Ghanem-Zoubi, 2024). The diseases it induces range from mild gastrointestinal manifestations (e.g., abdominal pain and diarrhea) to severe conditions such as skin and soft tissue infections, pneumonia, endocarditis, osteomyelitis, sepsis, and toxic shock syndrome (Zheng et al., 2025; Mlynarczyk-Bonikowska and Rudnicka, 2025; Tong et al., 2025). Consequently, in-depth investigation into the pathogenic mechanisms of *S. aureus* and the development of effective control strategies are crucial for safeguarding public health.

Currently, the clinical management of *S. aureus* infections predominantly relies on antibiotics, including β-lactams (e.g., methicillin, penicillin), glycopeptides (e.g., vancomycin), oxazolidinones (e.g., linezolid), and daptomycin (Tong et al., 2025; Liu et al., 2024). Nevertheless, the clinical application of these antibacterial agents is constrained by potential adverse effects, such as nephrotoxicity and bone marrow suppression (Wong et al., 2024; Karimzadeh et al., 2025). Consequently, there is an urgent need to develop alternative anti-*S. aureus* agents that combine high efficacy with low intrinsic toxicity, thereby reducing reliance on conventional antibiotics.

In this context, traditional Chinese medicine (TCM) offers a promising reservoir for the discovery of new anti-infective leads, owing to its advantages of multi-component, multi-target mechanisms, abundant natural sources, relatively favorable safety profiles, and a lower tendency to induce drug resistance (Deng et al., 2025; Liang et al., 2025). Many TCM formulations exhibit dual antibacterial and anti-inflammatory activities, highlighting their unique therapeutic potential (Tsai et al., 2013; Qin et al., 2026; Fernandes et al., 2025). *Clerodendrum cyrtophyllum* Turcz., a plant of the *Clerodendrum* genus (Verbenaceae family), is a commonly used ethnic medicinal herb in China. Traditionally, it has been employed for clearing heat, detoxification, and treating various infectious diseases due to its purported antibacterial and anti-inflammatory properties (Huang et al., 2025; Ran, 2017) Accumulating pharmacological evidence has revealed that its rich flavonoid constituents possess significant antibacterial, anti-inflammatory, and antioxidant activities (Pei et al., 2025; Nguyen et al., 2020), indicating considerable antimicrobial potential.

Despite this promise, systematic research on total flavonoids from *Clerodendrum cyrtophyllum* Turcz. (TFC) remains limited. To bridge this knowledge gap and facilitate the development of this botanical resource, the extraction conditions for TFC were optimized, and the crude TFC was purified using D101 macroporous resin. Subsequently, the chemical profile of the purified TFC was characterized by LC–MS/MS. Furthermore, the antibacterial activity and underlying mechanism of TFC against *S. aureus* were investigated. Collectively, these findings provide an experimental foundation to explore the medicinal potential of TFC against *S. aureus* and promote the development and utilization of *Clerodendrum cyrtophyllum* Turcz.

## 2 Materials and methods

### 2.1 Reagents

The following reagents and materials were used in this study. *Clerodendrum cyrtophyllum* Turcz. was collected from Baxian Village Wild Herbal Workshop and authenticated as the stems and leaves of this species by Prof. Xiangpei Wang, School of Ethnic Medicine, Guizhou Minzu University, Guiyang, China. The active compound TFC, isolated/purified from *Clerodendrum cyrtophyllum* Turcz., was dissolved in dimethyl sulfoxide (DMSO; Ruixin Chemical Co., Ltd., Shanghai, China) to prepare a 5% (v/v) stock solution. D101 macroporous resin, erythromycin, and 2′,7′-dichlorodihydrofluorescein diacetate (DCFH-DA) were purchased from Aladdin Biochemical Technology Co., Ltd. (Shanghai, China). Phosphate-buffered saline (PBS) was obtained from BKMAM Holdings Co., Ltd. (Hunan, China). LB agar plates were from Haibo Biotechnology Co., Ltd. (Qingdao, China). Rhodamine 123, *p*-nitrophenyl phosphate (pNPP), n-hexadecane, and rutin were supplied by Macklin Biochemical Co., Ltd. (Shanghai, China). 4′,6-diamidino-2-phenylindole (DAPI) was purchased from Lanjieke Technology Co., Ltd. (Beijing, China). A protein molecular weight marker was provided by Servicebio Technology Co., Ltd. (Wuhan, China). Unless otherwise stated, all chemicals and reagents were of analytical grade.

### 2.2 Bacterial strains and culture conditions

*S. aureus* used in this study was isolated from a dairy cow with clinical mastitis and characterized by molecular biology approaches (Pei et al., 2025). The isolate was stored in 20% glycerol at −80 °C until use. For each experiment, a glycerol stock was thawed, inoculated into Luria–Bertani (LB) broth, and cultured at 37 °C with agitation at 160 rpm for 18 h. An aliquot (100 μL) of this primary culture was then added to 10 mL of fresh LB broth and grown to mid-log phase (OD₆₀₀ ≈ 0.4–0.6). Cells were collected by centrifugation at 3500 × g for 5 min at 4 °C, washed twice with sterile phosphate-buffered saline (PBS), and resuspended in PBS for subsequent assays.

### 2.3 Determination of TFC content

The total flavonoid content was quantified by the aluminum nitrate-sodium nitrite colorimetric method (Tian et al., 2018), with rutin as the standard reference compound. Absorbance was measured at 510 nm using a UV–Vis spectrophotometer (model UV1802G, Tianjin Guanze Technology Co., Ltd., China), and a calibration curve was established as y = 10.275x + 0.0008 (*R*^2^ = 0.9995). After centrifugation of the *Clerodendrum cyrtophyllum* Turcz. extract (3,552 × g, 5 min), the supernatant was analyzed under the same colorimetric conditions. The TFC concentration (C) was calculated by substituting the absorbance value into the regression equation, and the extraction yield was determined using Equation 1:

$$\text{Extraction Rate}\;(\%)=\frac{C\times V\times N}{M}\times 100$$ (1)

where *C* is the mass concentration of total flavonoids in *Clerodendrum cyrtophyllum* Turcz (mg/mL); *V* is the volume of *Clerodendrum cyrtophyllum* Turcz. extract (mL); *N* is the dilution ratio; *M* is the mass of dry *Clerodendrum cyrtophyllum* Turcz. powder (mg).

### 2.4 BBD experimental design for TFC

#### 2.4.1 Single-factor experimental design

Exactly 1.0 g of *Clerodendrum cyrtophyllum* Turcz. powder was weighed and transferred into a 50 mL round-bottom flask. Ethanol was used as the extraction solvent, reflux extraction was performed under predetermined conditions of ethanol concentration, solid-to-liquid ratio, temperature, and extraction time. Immediately following extraction, the hot mixture was filtered under reduced pressure. The resulting filtrate was centrifuged at 3552 × g for 5 min, and the supernatant was collected for subsequent determination of TFC content. Details of the single-factor experimental design are provided in Table 1.

**Table 1:** Details of the single-factor experimental design.

Factor	Ethanol concentration (%)	Liquid-to-material ratio (g: ml)	Extraction temperature (°C)	Extraction time (min)
Ethanol concentration	50, 60, 70, 80, 90	1:20	60	60
Solid-to-liquid ratio	70	1:10, 1:15, 1:20, 1:25, 1:30	60	60
Extraction time	70	1:20	60	40, 60, 80, 100, 120
Extraction temperature	70	1:20	55, 65, 75, 85, 95	60

#### 2.4.2 Optimization of extraction variables using BBD method

Based on the promising ranges identified in the single-factor experiments, the central (zero) level for each factor was determined. The high and low levels were then set equidistantly around the central point, resulting in three coded levels (−1, 0, and +1) for each factor in the response surface methodology (RSM) design (Table 2). Using ethanol concentration, solid-to-liquid ratio, extraction temperature, and extraction time as independent variables and the TFC yield as the response, a four-factor, three-level Box–Behnken design (BBD) was generated using Design Expert software (Version 13.0, Stat-Ease Inc., Minneapolis, MN, USA). This design comprised 29 experimental runs, including five center points for estimating pure error. A quadratic polynomial regression model (Equation 2) was fitted to the experimental data. Analysis of variance (ANOVA) was used to assess the statistical significance of the model and its terms. Additionally, two-dimensional contour plots and three-dimensional response surface plots were generated to visualize the interactive effects of the variables on the TFC yield.

$$Y=\beta_{0}+\sum_{i=1}^{k}\beta_{i}X_{i}+\sum_{i=1}^{k}\beta_{\mathrm{ii}}X_{i}^{2}+\sum_{i=1}^{k}\sum_{j=1}^{k}\beta_{\mathrm{ij}}X_{i}X_{j}\;$$ (2)

**Table 2:** Factors and levels of response surface experiment.

Level	A: Ethanol concentration (%)	B: Solid-to-Liquid ratio (g: mL)	C: Extraction temperature (°C)	D: Extraction time (min)
−1	60	20	55	80
0	70	25	65	100
1	80	30	75	120

Where *Y* represents the measured response variables, *β*_0_ stands for the constant. *β_i_*, *β_ii_*, and *β_ij_* represent the linear, quadratic and interaction coefficient, respectively. *X_i_* and *X_j_* are the levels of independent variables, and k denotes the number of variables.

#### 2.4.3 Validation of optimal process conditions for TFC

The extraction of TFC using optimized independent extraction variables was carried out in triplicate (*n* = 3), and the experimental yield was compared with predicted values for model validation.

### 2.5 TFC purification

D101 macroporous adsorption resin was soaked in 95% (v/v) ethanol for 24 h to achieve sufficient full swelling. The resin was then sieved through a 60-mesh sieve and rinsed repeatedly with deionized water until the eluate became clear without any white turbidity. Subsequently, the resin was sequentially treated with 4% (v/v) HCl and 4% (w/v) NaOH solution for 4 h, followed by thorough rinsing with deionized water until the eluate reached neutrality (pH = 7.0). The pretreated resin was stored for subsequent use. Crude TFC prepared under the optimized extraction conditions were concentrated using a rotary evaporator, then redissolved in sterile distilled water to form a sample solution at a concentration of 2 mg/mL. The solution was loaded onto a chromatography column at a flow rate of 1.5 BV·h^−1^. After sample loading, the column was sequentially eluted with 4 bed volumes (BV) of distilled water and 3 BV of 50% (v/v) ethanol solution at a flow rate of 2 BV·h^−1^. The eluate was collected, concentrated under reduced pressure to dryness, yielding crude TFC powder. The crude TFC powder was mixed with 95% (v/v) ethanol at a solid-to-liquid ratio of 1:10 (g: mL), followed by ultrasonication at 100 W and 45 °C for 5 min. After complete dispersion and dissolution, the mixture was centrifuged at 3552 × g for 5 min. The supernatant was collected and evaporated to dryness under reduced pressure using a rotary evaporator, yielding a brownish-yellow TFC powder. The obtained TFC was stored in an amber, light-protected wide-mouth reagent bottle for subsequent experiments.

### 2.6 TFC purity analysis

The purity of TFC was calculated based on its absorbance measured according to Section 2.1. The absorbance was converted to concentration (C) via the rutin standard curve, and the purity was derived using Equation 3:

$$\text{Purity}(\%)=\frac{C\times V\times N}{m}\times 100$$ (3)

where *C* is the mass concentration of TFC in the purified assay solution (mg/mL); V is the final volume of the purified TFC solution after dilution to a fixed volume (mL); N is the dilution factor; m is the mass of the dried purified TFC (mg).

### 2.7 Analysis of TFC components

The analysis of TFC components was performed using LC–MS/MS, and all detection procedures were conducted by Beijing Keyangou Technology Co., Ltd. China. Briefly, 50 mg of TFC was extracted with 75% aqueous methanol via bead homogenization followed by 30 min of ultrasonication. After centrifugation at 17,000 × g for 10 min at 20 °C, the supernatant was filtered through a 0.22 μm PTFE syringe filter prior to LC–MS/MS analysis. Chromatographic separation was performed on an HSS T3 column (100 × 2.1 mm, 1.8 μm) maintained at 40 °C with a flow rate of 400 μL/min. The mobile phases consisted of water containing 0.1% formic acid (A) and acetonitrile containing 0.1% formic acid (B), with a 30-min gradient elution program (Table 3). Information-dependent acquisition (IDA) was employed for MS data collection, where the top 15 intense precursor ions (m/z range: 50–1,200; intensity threshold: >100 cps) were fragmented at a collision energy of 30 eV. Standard electrospray ionization (ESI) source parameters were applied as follows: source temperature, 550 °C; GS1/GS2 gas pressure, 60 psi; curtain gas pressure, 35 psi; spray voltage, +5,500 V (positive ion mode) and −4,500 V (negative ion mode). Raw LC–MS/MS data were converted to abf format using the Analysis Base File Converter software. Peak detection, peak alignment, and metabolite annotation were performed using MS-DIAL version 4.6. Tentative metabolite identification was achieved by matching experimental MS/MS spectra against public spectral libraries, including METLIN, MassBank (Japanese and European repositories), MoNA (MassBank of North America), and HMDB database (version 6.0). The key data processing parameters were set as follows: the accurate mass tolerances were 0.01 Da for MS and 0.05 Da for MS, with a cutoff identification score of 60%; the retention time tolerance was 0.2 min, and the MS mass tolerance for peak matching was 0.01 Da. Only compounds with positive dot product, reverse dot product, and fragment presence percentage values, together with a mass error of <5 ppm, were defined as high-confidence annotations.

**Table 3:** Mobile phase elution gradient.

Time (min)	Flow-rate (μL/min)	A (%)	B (%)
0.00	400	95	5
1.50	400	95	5
2.50	400	90	10
14.00	400	60	40
24.00	400	5	95
27.00	400	5	95
27.10	400	95	5
30.00	400	95	5

Mobile phase A: water with 0.1% formic acid; Mobile phase B: acetonitrile with 0.1% formic acid.

### 2.8 Antimicrobial activity

#### 2.8.1 Measurement of bacteriostatic zone diameter

The bacteriostatic zone diameter of TFC was evaluated by the Oxford cup method (Wang L. et al., 2025). *S. aureus* cultures in the logarithmic growth phase were diluted 100-fold with sterile distilled water. Then, 100 μL of the diluted bacterial suspension was evenly spread onto the surface of an LB nutrient agar plate using a sterile spreader. Three sterile Oxford cups were placed equidistantly on the inoculated plate and loaded with 150 μL of TFC sample solutions at 50 mg/mL, 100 mg/mL, and 150 mg/mL, respectively. An equal volume of DMSO was used as the negative control, and erythromycin (100 μg/mL) served as the positive control. After incubation at 37 °C for 24 h, the diameters of the transparent inhibition zones around each cup were precisely measured. Data are presented as mean ± standard deviation.

#### 2.8.2 Determination of MIC and MBC

MIC Determination: The MIC of TFC was determined using a standard two-fold broth microdilution method (Kashyap et al., 2025). Briefly, TFC was serially diluted in sterile LB broth in a 96-well plate to final concentrations of 25, 12.5, 6.25, 3.125, 1.562, 0.781, 0.390, 0.195, and 0.097 mg/mL. Each well contained 100 μL of the diluted TFC solution. A logarithmic-phase culture of *S. aureus* was adjusted to 1.0 × 10^7^ CFU/mL in LB broth, and 100 μL of this bacterial suspension was added to each well. Wells containing erythromycin (positive control), an equal volume of DMSO (negative control), and sterile LB broth (blank control) were included. The plate was incubated at 37 °C for 24 h, after which turbidity in each well was assessed visually. The MIC was recorded as the lowest concentration of TFC that completely inhibited visible bacterial growth.

MBC Determination: Based on the results of the MIC assay, 100 μL of the culture broth from the clear well (indicating no visible growth) and the well corresponding to 1/2 × MIC was collected, evenly spread on LB nutrient agar plates, and incubated at 37 °C for 24 h. The MBC was defined as the lowest sample concentration that resulted in a ≥ 99.9% reduction in colony count on the agar plates. In general, a sample is considered bactericidal if the MBC value is no greater than four times its MIC value (Barry and Lasner, 1979).

#### 2.8.3 Growth curve

A logarithmic-phase *S. aureus* suspension was adjusted to 1.0 × 10^7^ CFU/mL. TFC was added to *S. aureus* suspension at final concentrations of 1/2 × MIC, 1 × MIC, and 2 × MIC, while a group without TFC served as the blank control. The cultures were incubated at 37 °C with shaking at 150 rpm for 24 h. Samples were collected every 2 h, and the optical density at 600 nm (OD₆₀₀) was measured. Growth curves for *S. aureus* were plotted using GraphPad Prism 8.0, with time as the x-axis and OD₆₀₀ as the y-axis.

### 2.9 Antibacterial mechanism

#### 2.9.1 Cell surface hydrophobicity test

The cell surface hydrophobicity (CSH) of *S. aureus* was evaluated by the microbial adhesion to hydrocarbons (MATH) assay as previously described (Evans et al., 2022). Briefly, *S. aureus* was treated as outlined in Section 2.2. TFC was added to *S. aureus* suspension at final concentrations of 1/2 × MIC, 1 × MIC, and 2 × MIC, while *S. aureus* suspension without TFC served as an untreated control. After incubation at 37 °C for 4 h, 1 mL of each culture was transferred to a clean tube, and its OD₆₀₀ was measured and recorded as A₀. Then, 400 μL of n-hexadecane was accurately added to each tube. The mixture was vigorously vortexed for 1 min to ensure thorough contact between the bacterial cells and the hydrocarbon phase, and then incubated at 37 °C for 15 min to achieve complete phase separation. After stable stratification, the OD₆₀₀ of the lower aqueous phase was measured and defined as A. The CSH, expressed as the bacterial adhesion rate to n-hexadecane, was calculated using Equation 4:

$$\mathrm{CSH}(\%)=\frac{\mathrm{Ao}-A}{\mathrm{Ao}}\times 100$$ (4)

Where A₀ is the OD_600_ value of the bacterial suspension prior to mixing with n-hexadecane, and A is the OD_600_ value of the aqueous phase after mixing with n-hexadecane and allowing separation by settling.

#### 2.9.2 Measurement of TFC on alkaline phosphatase (AKP) activity of *S. aureus*

AKP activity was measured using the pNPP assay as previously described (Pei et al., 2025). *S. aureus* was treated as outlined in Section 2.2. Bacterial suspensions were supplemented with TFC to achieve final concentrations of 1/2 × MIC, 1 × MIC, and 2 × MIC. Samples without TFC treatment served as the untreated control. All samples were incubated at 37 °C with shaking at 130 rpm. At 0.5 h intervals (up to 3 h), 3 mL aliquots were withdrawn and centrifuged at 3000 × *g* for 10 min. The supernatants were collected and mixed with 0.3 mL of 1 mg/mL pNPP solution. After gentle vortexing, the mixtures were incubated in the dark at 37 °C for 30 min. The reaction was terminated by adding 0.12 mL of 1 mol/L NaOH. The absorbance at 405 nm (OD₄₀₅) was measured using a UV–Vis spectrophotometer. All experiments were performed in triplicate.

#### 2.9.3 Membrane permeability assays

The effect of TFC on the membrane permeability of *S. aureus* was evaluated by quantifying the release of protein and nucleic acid leakage. *S. aureus* was treated as outlined in Section 2.2. Bacterial suspensions were supplemented with TFC to achieve final concentrations of 1/2 × MIC, 1 × MIC, and 2 × MIC. Samples without TFC treatment served as the untreated control. All samples were incubated in a shaking incubator at 37 °C and 130 rpm for 6 h. Aliquots were collected at 1.5-h intervals and centrifuged at 1400 × *g* for 10 min at 4 °C to prepare cell-free supernatants. The absorbance of each supernatant at 260 nm (OD_260_) and 280 nm (OD_280_) was measured using a UV–Vis spectrophotometer to assess the levels of released nucleic acids and proteins, respectively. All measurements were performed in triplicate.

#### 2.9.4 Measurement of bacterial membrane potential

To assess the effect of TFC on bacterial membrane integrity and energy metabolism, changes in the membrane potential of *S. aureus* were determined using the fluorescent probe Rhodamine 123 as described previously (Wang Y. et al., 2025). Briefly, *S. aureus* was treated as outlined in Section 2.2. Bacterial suspensions were supplemented with TFC to achieve final concentrations of 1/2 × MIC, 1 × MIC, and 2 × MIC. Samples without TFC treatment served as the untreated control. Rhodamine 123 stock solution (1 mg/mL) was added to each sample to a final concentration of 2 μg/mL. After incubation in the dark at 37 °C for 30 min to allow dye uptake, the cells were pelleted by centrifugation (1,400 × *g*, 5 min, 4 °C) and washed twice with PBS to remove unbound dye. The stained cell pellets were resuspended in PBS and transferred to a black 96-well plate. Fluorescence intensity was measured using a microplate reader with excitation and emission wavelengths set at 480 nm and 530 nm, respectively. All experiments were performed in triplicate.

#### 2.9.5 Intracellular nucleic acids assay

The extent of membrane permeability disruption induced by TFC was further assessed by measuring the fluorescence intensity of DAPI bound to intracellular nucleic acids (Gildea et al., 2022). *S. aureus* was treated as outlined in Section 2.2. Bacterial suspensions were supplemented with TFC to achieve final concentrations of 1/2 × MIC, 1 × MIC, and 2 × MIC. Samples without TFC treatment served as the untreated control. Following incubation at 37 °C with shaking (180 rpm) for 24 h, the cells were collected by centrifugation at 5590 × *g* for 5 min, washed three times with PBS, and resuspended. A 0.4 mL aliquot of the cell suspension was mixed with 1.2 mL of DAPI staining solution to achieve a final concentration of 0.8 μg/mL. After incubation in the dark at 37 °C with gentle shaking for 10 min, fluorescence intensities for DAPI-DNA complexes (λex = 364 nm, λem = 460 nm) and DAPI-RNA complexes (λex = 400 nm, λem = 500 nm) were measured using a microplate reader. All experiments were performed in triplicate.

#### 2.9.6 Intracellular protein assay

Sodium dodecyl sulfate–polyacrylamide gel electrophoresis (SDS-PAGE) was performed to evaluate the effect of TFC on the total protein profile of *S. aureus*. *S. aureus* isolate was treated as outlined in Section 2.2 and cultured to mid-log phase. *S. aureus* suspensions were supplemented with TFC to achieve final concentrations of 1/2 × MIC, 1 × MIC, and 2 × MIC; samples without TFC treatment served as the untreated control. All cultures were incubated with shaking (150 rpm) at 37 °C. Aliquots were harvested at 4, 16, and 24 h, and cells were pelleted by centrifugation at 5,600 × g for 5 min at 4 °C. The pellets were washed twice with sterile PBS, resuspended in 50 μL of sterile deionized water and 200 μL of 2 × Laemmli reducing sample buffer (125 mM Tris–HCl pH 6.8, 4% (w/v) SDS, 20% (v/v) glycerol, 0.02% (w/v) bromophenol blue, 10% (v/v) β-mercaptoethanol), and vortexed vigorously. After boiling at 100 °C for 10 min, samples were centrifuged at 8,000 × g for 10 min to remove debris. For electrophoretic separation, the supernatant (15 μL per lane) was loaded onto a 5% stacking gel over a 12% separating gel using a Laemmli discontinuous buffer system. The gel was run in 1 × Tris–glycine–SDS running buffer (25 mM Tris, 192 mM glycine, 0.1% (w/v) SDS, pH 8.3) under constant voltage: 80 V through the stacking gel, then increased to 120 V after the bromophenol blue front entered the separating gel, until the dye front reached the bottom. After electrophoresis, the gel was fixed in 50% methanol / 10% acetic acid for 30 min, stained for 4 h in 0.1% Coomassie Brilliant Blue R-250 dissolved in 45% methanol / 10% acetic acid, and destained in 10% methanol / 10% acetic acid with several changes until clear background was obtained. Gel images were captured using a gel documentation system.

#### 2.9.7 Determination of reactive oxygen species (ROS)

The intracellular ROS levels in *S. aureus* following TFC treatment were dynamically monitored using the fluorescent probe DCFH-DA (Shivaprasad et al., 2021). *S. aureus* was treated as outlined in Section 2.2. The bacterial suspension was then incubated with 1 μL DCFH-DA for 20 min at 37 °C in the dark. After incubation, the cells were pelleted by centrifugation (1,400 × *g*, 5 min, 4 °C) and washed twice with PBS to thoroughly remove extracellular dye. The stained cells were resuspended in fresh PBS. Subsequently, TFC was added to achieve final concentrations of 1/2 × MIC, 1 × MIC, and 2 × MIC. A sample without TFC served as the untreated control. Aliquots of each treatment were transferred to a black 96-well microplate and incubated at 37 °C. Fluorescence intensity was measured every 30 min using a microplate reader at an excitation wavelength of 488 nm and emission wavelength of 525 nm. All experiments were performed with three independent biological replicates.

#### 2.9.8 Scanning electron microscopy (SEM) assay

SEM was performed following the method described by Wan et al. (2021) with slight modifications and completed by Chengdu Lilai Biotechnology Co., Ltd. China. Briefly, mid-logarithmic-phase *S. aureus* suspensions were prepared as outlined in Section 2.2 and treated with TFC at final concentrations of 0 × MIC, 1 × MIC, and 2 × MIC. After incubation at 37 °C with shaking at 130 rpm for 6 h or 12 h, bacterial cells were harvested by centrifugation at 5000 × *g* for 5 min. The pellets were washed twice with PBS and fixed overnight with 2% glutaraldehyde at 4 °C. Following three rinses with PBS, samples were post-fixed with 1% osmium tetroxide (OsO₄) in the dark for 2 h. The fixed samples were then washed three times with ultrapure water (10 min per wash) and dehydrated through a graded ethanol series (30, 50, 70, 90, and 100%; three changes of 100% ethanol), with each step lasting 15 min. An aliquot of the sample suspension was dropped onto a sterile glass coverslip, followed by critical point drying with CO₂. The dried coverslips were mounted onto specimen stubs using conductive adhesive, sputter-coated with gold using an ion sputter coater, and examined using a field-emission scanning electron microscope for morphological examination and image acquisition.

#### 2.9.9 Determination of intracellular adenosine triphosphate (ATP) concentrations

Intracellular ATP levels in *S. aureus* were determined according to Luo et al. (2022). Briefly, mid-logarithmic-phase *S. aureus* suspensions (1.0 × 10^7^ CFU/mL) were treated with TFC at final concentrations of 1/2 × MIC, 1 × MIC, or 2 × MIC, or left untreated as a control. After incubation at 37 °C with shaking at 130 rpm for 30 min, bacterial cells were harvested by centrifugation at 5000 × *g* for 5 min. Intracellular ATP concentrations were then measured using a commercial ATP Assay Kit (Nanjing Jiancheng Bioengineering Institute, Nanjing, China) according to the manufacturer’s instructions.

### 2.10 Statistical analysis

All experimental data were preprocessed and expressed as mean ± standard deviation (SD), with error bars representing SD. Statistical analyses were performed using GraphPad Prism 8.0 software. Comparisons among multiple groups were conducted using one-way analysis of variance ANOVA. All experiments were performed independently in at least three biological replicates. Statistical significance was defined as follows: *p < 0.05, **p < 0.01, and ***p < 0.001.

## 3 Results

### 3.1 BBD experimental design for TFC

#### 3.1.1 Results of single-factor experiment

The effects of four independent extraction parameters on TFC yield are presented in Figure 1. The extraction yield of TFC as a function of four independent parameters is shown in Figure 1. The yield increased with increasing ethanol concentration from 50 to 70%, reaching a maximum of approximately 4.9% at 70% (Figure 1A). Beyond this optimum, a further increase to 90% led to a gradual decline in yield to 4.2%; Varying the solid-to-liquid ratio significantly affected the extraction efficiency (Figure 1B). The yield steadily increased as the ratio changed from 1:10 to 1:25 (g: mL), attaining an optimum of about 4.9% at 1:25. A further increase to 1:35 resulted in a slight reduction in yield to roughly 4.7%; Extraction temperature also showed a pronounced effect (Figure 1C). The yield rose sharply from 55 °C to 65 °C, peaking at 4.79%. A subsequent increase to 100 °C caused a consistent decrease, with the yield falling to approximately 4.4%; Similarly, extraction time markedly influenced the yield (Figure 1D). An increase from 40 min to 100 min raised the yield from 3.6% to a maximum of 4.82%. However, extending the extraction to 120 min reduced the yield to 4.6%.

**Figure 1:**
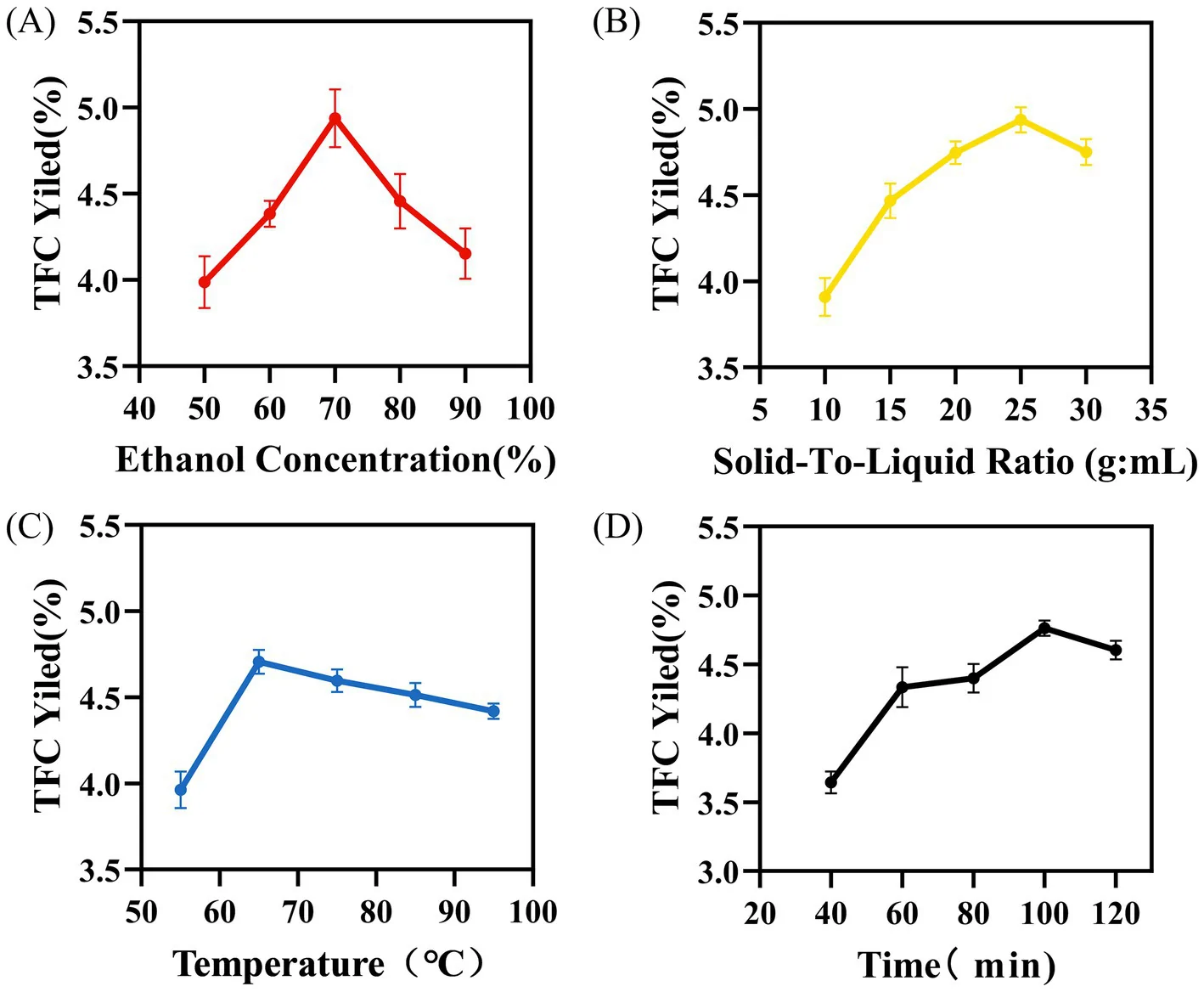
Effects of ethanol concentration, solid–liquid ratio, temperature, and extraction time on the yield of TFC. **(A)** Ethanol concentration; **(B)** Solid-to-liquid ratio; **(C)** Temperature; **(D)** Time.

Based on these results, the following preliminary optimal conditions were identified: ethanol concentration of 70%, solid-to-liquid ratio of 1:25 (g: mL), extraction temperature of 65 °C, and extraction time of 100 min. These conditions defined the central points for factor level selection in subsequent RSM optimization experiments. The aforementioned ~4.9% maxima under individual single-factor elevation represent isolated baseline yields without multivariate synergy, and thus cannot reach the coordinated optimum revealed by the subsequent RSM-BBD model.

#### 3.1.2 Response surface optimization results and analysis

##### 3.1.2.1 Model development and analysis of variance

Based on the levels and factors defined in Table 2, the 29 experimental runs designed by RSM-BBD and their corresponding results are presented in Table 4. The results were analyzed using Design-Expert 13 software. After regression fitting, the following quadratic polynomial regression equation was obtained:

$$\begin{array}{l} Y=6.528+0.146667A+0.108333B-0.259167C+0.1675D\\ -0.04\mathrm{5AB}+0.2\;\mathrm{AC}+0.12\;\mathrm{AD}+0.255\;\mathrm{BC}-0.10\mathrm{5BD}\\ -0.2425\mathrm{CD}-1.1119\mathrm{2A}2–1.0944\mathrm{2B}2–1.1131\mathrm{7C}2–0.850667\mathrm{D2}. \end{array}$$

**Table 4:** Response surface experiment results.

No.	A: ethanol concentration (%)	B: solid-to-liquid ratio (g: mL)	C: extraction temperature (°C)	D: extraction time (min)	TFC yield (%)
1	80	20	65	100	4.38
2	80	25	75	100	4.42
3	80	25	55	100	4.52
4	70	20	55	100	4.77
5	70	20	65	80	4.2
6	60	25	65	80	4.42
7	60	25	55	100	4.59
8	70	25	65	100	6.68
9	70	30	75	100	4.43
10	70	20	75	100	3.7
11	60	20	65	100	3.99
12	60	25	75	100	3.69
13	70	25	65	100	6.5
14	60	30	65	100	4.3
15	70	25	65	100	6.59
16	70	20	65	120	4.76
17	70	30	65	80	4.62
18	80	30	65	100	4.51
19	70	30	55	100	4.48
20	70	30	65	120	4.76
21	60	25	65	120	4.51
22	80	25	65	120	5
23	80	25	65	80	4.43
24	70	25	55	80	4.38
25	70	25	75	120	4.21
26	70	25	65	100	6.42
27	70	25	75	80	4.37
28	70	25	55	120	5.19
29	70	25	65	100	6.45

The variance and significance analysis results of the above regression model are shown in Table 5. ANOVA was performed on the established quadratic polynomial model to assess its significance. The *F*-value and *p*-value were used to determine whether the regression model is statistically significant. Meanwhile, the lack-of-fit test was conducted to examine whether the model omitted systematic errors or important structural terms. In general, a larger *F*-value and a smaller *p*-value for a term indicate a more significant effect on TFC yield. The *F*-value of the present model is 328.00 with a *p*-value of < 0.001, demonstrating that the regression model is highly significant overall and can effectively explain the relationship between the factors and the response. The lack-of-fit *F*-value is 0.1462 with a *p*-value of 0.9936 (> 0.05), which is not significant, indicating that the model fits the data well and the error is mainly due to random variation rather than systematic bias. The coefficient of determination *R*^2^ is 0.9970, meaning that the model explains 99.70% of the variation in the response, reflecting an excellent fit. The adjusted *R*^2^ (Adj-*R*^2^) is 0.9939, and the predicted *R*^2^ (Pred-*R*^2^) is 0.9918. The close agreement between Adj-*R*^2^ and Pred-*R*^2^, with a difference of less than 0.2, suggests that the model possesses high explanatory power and good predictive capability. The adequate precision ratio is 58.6465, which is well above the acceptable threshold of 4, confirming that the model provides sufficient signal for reliable analysis and navigation within the design space. Significance analysis of individual terms reveals that the linear terms A (ethanol concentration), B (solid-to-liquid ratio), C (temperature), and D (extraction time), the interaction terms AC, AD, BC, BD, and CD, and all quadratic terms A^2^, B^2^, C^2^, and D^2^ have significant effects on the response (TFC yield) (*p* < 0.05). The interaction term AB is not significant (*p* = 0.2010 > 0.05). Based on the *F*-values, the order of influence of the factors on the yield is: C (extraction temperature) > D (extraction time) > A (ethanol concentration) > B (solid-to-liquid ratio).

**Table 5:** ANOVA results of the regression model.

Source	Sum of squares	df	Mean square	*F*-value	*P*-value	Significance notation
Model	20.66	14	1.48	328.00	< 0.0001	***
A	0.2581	1	0.2581	57.38	< 0.0001	***
B	0.1408	1	0.1408	31.31	< 0.0001	***
C	0.8060	1	0.8060	179.17	< 0.0001	***
D	0.3367	1	0.3367	74.84	< 0.0001	***
AB	0.0081	1	0.0081	1.80	0.2010	ns
AC	0.1600	1	0.1600	35.57	< 0.0001	***
AD	0.0576	1	0.0576	12.80	0.0030	**
BC	0.2601	1	0.2601	57.82	< 0.0001	***
BD	0.0441	1	0.0441	9.80	0.0074	**
CD	0.2352	1	0.2352	52.29	< 0.0001	***
A^2^	8.01	1	8.01	1779.55	< 0.0001	***
B^2^	7.75	1	7.75	1723.92	< 0.0001	***
C^2^	8.02	1	8.02	1783.55	< 0.0001	***
D^2^	4.68	1	4.68	1040.98	< 0.0001	***
Residual	0.0630	14	0.0045			
Lack of fit	0.0169	10	0.0017	0.1462	0.9936	ns
Pure error	0.0461	4	0.0115			
Cor total	20.72	28				
*R*^2^	0.9970					
Adj. *R*^2^	0.9939					
Pred-*R*^2^	0.9918					
Adeq precision	58.6465					

**, indicates significant (*p* < 0.01); ***, indicates highly significant (*p* < 0.001); ns, indicates not significant (*p* ≥ 0.05).

##### 3.1.2.2 Graphical analysis of factor interactions

In response surface methodology, response surface plots and contour plots serve as essential visualization tools, which can intuitively illustrate the effects of individual factors on TFC yield and their interactions. The geometric shape of the response surface, as well as the steepness and curvature of the surface, reflect the strength of each variable’s influence on the response and the significance of their interactive effects. A steeper slope of the response surface along a given factor direction indicates a greater influence of that factor on TFC yield, namely a more significant main effect; in contrast, a gentle slope suggests a weaker effect. Analysis of the three-dimensional response surface plots reveals that the overall surface exhibits a downward-opening, convex “hill-like” structure, indicating the presence of a distinct maximum point for the response within the experimental design range. Meanwhile, the contour plot, as a two-dimensional projection of the three-dimensional response surface, clearly and visually expresses the interaction between variables. When the contour lines show a distinct elliptical shape, it suggests a significant interaction between the corresponding two factors; conversely, near-circular contours imply a nonsignificant interaction.

As shown in Figure 2, comparative analysis of the six sets of three-dimensional response surfaces and their corresponding contour plots indicates that the interaction effects are more pronounced for the pairs AC (B1), AD (C1), BC (D1), BD (E1), and CD (F1). These five interaction groups exhibit noticeable curvature changes in the response surfaces, suggesting that their interactive effects have a significant impact on TFC yield. The corresponding contour plots (B2, C2, D2, E2, F2) display clear elliptical shapes, further confirming from a geometric perspective that significant interactions exist between these factors. In contrast, the response surface for the AB pair (A1) is relatively flat with little undulation, and its corresponding contour plot (A2) is nearly circular, reflecting a weaker interaction between these two factors and a relatively limited influence on TFC yield. The visual interpretations based on the morphological features of the response surfaces and contour plots are consistent with the significance test results of the interaction terms in the ANOVA. Together, they demonstrate that within the selected experimental design range, the influences of different factors on the TFC extraction process vary considerably, with the interaction terms AC, AD, BC, BD, and CD being particularly prominent, thereby providing clear priority directions for subsequent process optimization.

**Figure 2:**
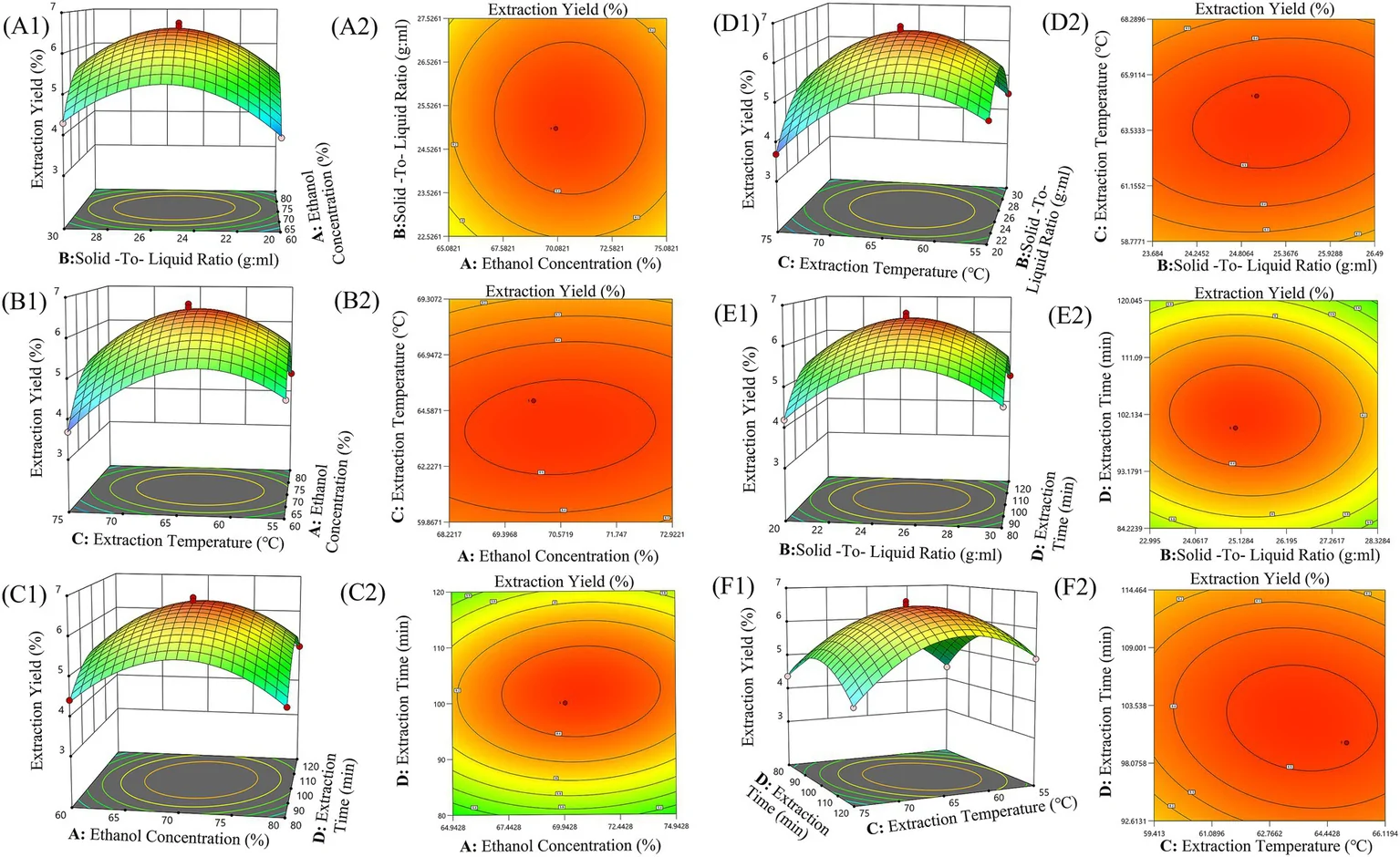
Three-dimensional response surfaces and two-dimensional contour maps depicting the interactive effects of factors on TFC yield. **(A)** Ethanol concentration and solid-to-liquid ratio; **(B)** extraction temperature and ethanol concentration; **(C)** ethanol concentration and extraction time; **(D)** extraction temperature and solid-to-liquid ratio; **(E)** extraction time and solid-to-liquid ratio; **(F)** extraction time and extraction temperature.

##### 3.1.2.3 Validation of the response surface model

The normal probability plot of residuals for the response surface model (Figure 3A) shows that the externally studentized residuals generally follow the theoretical normal distribution line, with only minor outliers present. This indicates that the model residuals satisfy the assumption of normality, and the systematic error is random without significant model misspecification. In the predicted versus actual values scatter plot (Figure 3B), the data points are closely distributed along both sides of the diagonal line (y = x), demonstrating high consistency especially within the main response range (TFC yield approximately 3 to 7%). This confirms that the model fits the experimental data well and exhibits high predictive accuracy. Together, these results validate the statistical soundness and predictive reliability of the response surface model developed in this study.

**Figure 3:**
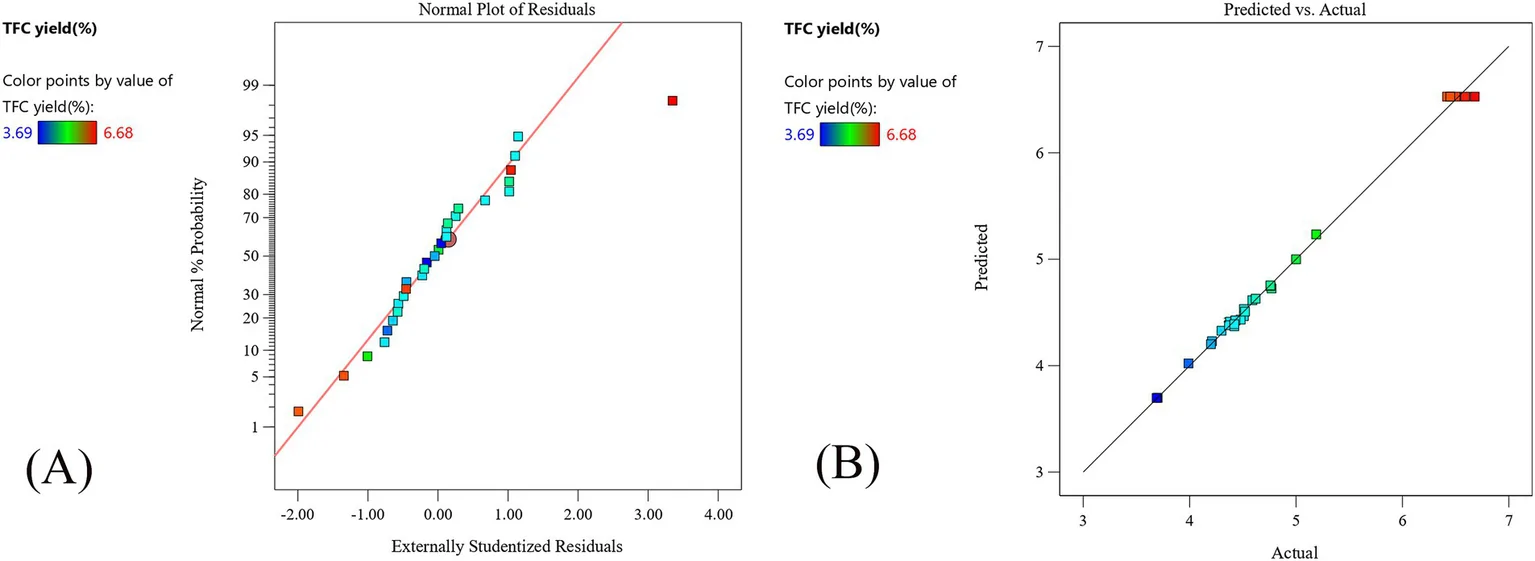
Diagnostic plots for the regression model of TFC yield. **(A)** Normal probability plot of residuals; **(B)** Predicted vs. actual values scatter plot.

##### 3.1.2.4 Validation of optimal process conditions for TFC

Based on the analysis performed with Design Expert 13 software, the predicted optimum extraction conditions for TFC were determined as follows: ethanol concentration 70.6147%, extraction temperature 63.7985 °C, solid-to-liquid ratio 1:25.1408 (g: mL), and extraction time 102.362 min. Under these conditions, the predicted TFC extraction yield was 6.55953%. To ensure practical operability in the laboratory, the above conditions were slightly adjusted to: ethanol concentration 70%, extraction temperature 64 °C, solid-to-liquid ratio 1:25 (g: mL), and extraction time 102 min. Three independent verification experiments conducted under the adjusted conditions yielded an average TFC extraction rate of 6.48% ± 0.58, which showed no significant difference from the predicted value. This result indicates that the regression model fits the experimental data well and that the optimized conditions are reliable.

#### 3.1.3 Purity evaluation

The final product obtained through the aforementioned procedure and purified by D101 macroporous resin. A standard calibration curve was constructed using rutin, yielding the linear equation (Y = 10.275X + 0.0008, *R*^2^ = 0.9994). Based on this curve, the maximum extraction yield of TFC was calculated to be 6.48% ± 0.58, with a purity of 76%. These results demonstrate that the purification process established in this study enables the efficient enrichment of flavonoids with high purity from *Clerodendrum cyrtophyllum* Turcz., making the obtained TFC suitable for subsequent bioactivity investigations and pharmaceutical development.

### 3.2 Identification of flavonoid compounds of TFC by LC–MS/MS

The components of TFC were identified via LC–MS/MS. A total of 35 and 60 flavonoids were separately identified from TFC in negative and positive ESI mode, respectively (Tables 6, 7). Notably, 10 components, including andrographidin B, demethoxycentaureidin 7-O-rutinoside, jaceoside, neodiosmin, cirsimaritin, isorhamnetin 3-galactoside, pectolinarin, velutin, iridin, and chrysoeriol, were consistently detected in both ESI modes. Collectively, a total of 85 flavonoid components were identified in TFC. The major abundant flavonoid components of TFC, reported as relative peak area abundance rather than absolute quantification, included andrographidin B (22.78%), citrusunshin A (16.00%), eupatilin (7.86%), jacein (5.80%), tricin methyl ether (5.20%), and rhamnazin (4.66%), with these values expressed as the percentage of each component’s peak area relative to the total integrated peak area of all identified flavonoids in the TIC. Representative total ion chromatograms under positive- and negative-ion modes, together with the MS/MS spectra of the six aforementioned flavonoids, are presented in Figure 4, where the six aforementioned flavonoids are labelled near their approximate chromatographic peak regions.

**Table 6:** Negative ESI-MS/MS identification of flavonoids in purified TFC.

No.	Title	RT (min)	Formula	Precursor m/z	Theoretical m/z	PPM	Major fragment ions
1	Vicenin 2	6.86	C_27_H_30_O_15_	593.1511	593.1512	0.1669	593.1534
2	Tomentosanol A	5.98	C_26_H_30_O_6_	437.1971	437.1970	0.3111	135.0264
3	Gossypin	6.90	C_21_H_20_O_13_	479.0833	479.0831	0.3778	161.0237
4	Maritimein	10.59	C_21_H_20_O_11_	447.0931	447.0933	0.4227	285.0402
5	Pectolinarin	10.03	C_29_H_34_O_15_	621.1829	621.1825	0.6455	621.1856
6	Cirsiliol	14.98	C_17_H_14_O_7_	329.0669	329.0667	0.6716	299.0194
7	Tectoridin	10.38	C_22_H_22_O_11_	461.1086	461.1089	0.7352	461.1103
8	Jaceidin	15.40	C_18_H_16_O_8_	359.0768	359.0772	1.2365	329.0328
9	IRIDIN	11.53	C_24_H_26_O_13_	521.1294	521.1301	1.2837	344.0529
10	Demethoxycentaureidin 7-O-rutinoside	11.67	C_29_H_34_O_16_	637.1765	637.1774	1.4345	343.0825
11	Lepidoside	14.52	C_26_H_28_O_14_	563.1398	563.1406	1.4810	313.0755
12	Velutin	14.75	C_17_H_14_O_6_	313.0713	313.0718	1.4821	255.0306
13	Gnaphaliin	11.99	C_17_H_14_O_6_	313.0713	313.0718	1.4821	283.025
14	Thermopsoside, crotonoyl	14.56	C_26_H_26_O_12_	529.136	529.1352	1.5988	529.1413
15	skullcapflavone II	17.02	C_19_H_18_O_8_	373.0935	373.0929	1.6243	343.0437
16	Odoratin	18.36	C_17_H_14_O_6_	313.0712	313.0718	1.8015	255.0328
17	Isorhamnetin 3-galactoside	11.09	C_22_H_22_O_12_	477.1029	477.1039	1.9996	315.0499
18	eriocitrin	6.27	C_27_H_32_O_15_	595.1656	595.1668	2.0986	595.1676
19	Neoliquiritin	1.16	C_21_H_22_O_9_	417.1182	417.1191	2.1792	417.1002
20	Dihydrohesperetin-7-O-neohesperidoside	9.23	C_28_H_36_O_15_	611.1968	611.1981	2.2071	197.0543
21	Jaceoside	10.63	C_23_H_24_O_12_	491.1208	491.1195	2.6389	491.1196
22	Quercetin 3-(2-xylosylrhamnoside)	8.95	C_26_H_28_O_15_	579.1339	579.1355	2.8473	417.1054
23	Neodiosmin	8.39	C_28_H_32_O_15_	607.1686	607.1668	2.8839	225.0814
24	Cirsimaritin	16.76	C_17_H_14_O_6_	313.0708	313.0718	3.0792	283.0248
25	Kaempferol 3-O-sophoroside	8.29	C_27_H_30_O_16_	609.1442	609.1461	3.1421	447.1156
26	Andrographidin B	11.83	C_23_H_24_O_12_	491.1211	491.1195	3.2497	329.0689
27	Citrusunshin A	12.30	C_25_H_28_O_14_	551.1425	551.1406	3.3857	343.0856
28	Eupatilin	12.30	C_18_H_16_O_7_	343.0835	343.0823	3.4132	313.0354
29	Kaempferol-3-O-galactoside-7-O-rhamnoside	9.29	C_27_H_30_O_15_	593.1534	593.1512	3.7107	447.1152
30	Chrysoeriol	11.55	C_16_H_12_O_6_	299.055	299.0561	3.7251	284.0316
31	7-GlcA tricin	10.69	C_23_H_22_O_13_	505.1008	505.0988	4.0210	314.0418
32	Puerariaglycoside 3	13.71	C_22_H_22_O_10_	445.1161	445.1140	4.6640	152.0129
33	Cynaroside	8.98	C_21_H_20_O_11_	447.0912	447.0933	4.6724	285.0431
34	Isorhamnetin-3-O-rutinoside	6.40	C_28_H_32_O_16_	623.1647	623.1618	4.7115	579.1694
35	Luteolin	12.63	C_15_H_10_O_6_	285.0391	285.0405	4.7853	133.0297

**Table 7:** Positive ESI-MS/MS identification of flavonoids in purified TFC.

No.	Title	RT (min)	Formula	Precursor m/z	Theoretical m/z	PPM	Major fragment ions
1	Baicalin	10.10	C_21_H_18_O_11_	447.0922	447.0922	0.0201	271.06233
2	Isoshaftoside	12.62	C_26_H_28_O_14_	565.1552	565.1552	0.0248	345.09894
3	3-Hydroxy-6,3′,4′-trimethoxyflavone	12.69	C_18_H_16_O_6_	329.1020	329.1020	0.1033	329.10243
4	Epimedin C	12.92	C_39_H_50_O_19_	823.3019	823.3020	0.1081	823.30449
5	Glabranin	22.27	C_20_H_20_O_4_	325.1434	325.1434	0.1107	163.04168
6	Acacetin-7-O-neohesperidoside	13.88	C_28_H_32_O_14_	593.1865	593.1866	0.1922	345.09677
7	3-Hydroxy-7,8,3′-trimethoxyflavone	19.85	C_18_H_16_O_6_	329.1020	329.1019	0.2005	329.10222
8	Jaceoside	9.24	C_23_H_24_O_12_	493.1341	493.1342	0.2920	331.08255
9	Capreoside	9.95	C_27_H_30_O_15_	595.1658	595.1655	0.4217	301.07209
10	5-Hydroxy-2′,4′,7,8-Tetramethoxyflavone	18.75	C_19_H_18_O_7_	359.1125	359.1127	0.4706	359.11342
11	Spinosin B	12.60	C_38_H_40_O_18_	785.2287	785.2283	0.5680	345.09643
12	Pectolinarigenin	13.58	C_17_H_14_O_6_	315.0863	315.0865	0.5840	315.08201
13	Pectolinarin	8.52	C_29_H_34_O_15_	623.1971	623.1975	0.7205	147.04497
14	Axillarin	10.90	C_17_H_14_O_8_	347.0761	347.0764	0.7318	347.10583
15	(.+/−.)-Medicarpin	7.40	C_16_H_14_O_4_	271.0965	271.0967	0.7894	165.07201
16	Hesperidin methyl chalcone	8.43	C_29_H_36_O_15_	625.2127	625.2132	0.7981	147.04507
17	haploside D	10.86	C_30_H_34_O_18_	683.1818	683.1824	0.8841	345.09808
18	(−)-Epigallocatechin	9.39	C_15_H_14_O_7_	307.0812	307.0809	1.0779	163.03958
19	3,5,6,7,8,3′,4’-Heptamethoxyflavone	4.58	C_22_H_24_O_9_	433.1493	433.1488	1.1797	433.15262
20	Baicalin methyl ester	8.70	C_22_H_20_O_11_	461.1078	461.1084	1.2123	163.0397
21	Cirsimaritin	11.88	C_17_H_14_O_6_	315.0863	315.0867	1.2187	315.08969
22	Isorhamnetin 3-galactoside	11.02	C_22_H_22_O_12_	479.1184	479.1178	1.2648	317.06748
23	Dehydrodiisoeugenol	8.09	C_20_H_22_O_4_	327.1591	327.1595	1.2654	163.03603
24	Licoflavone A	13.67	C_20_H_18_O_4_	323.1278	323.1273	1.5040	291.1018
25	Chrysoeriol	14.62	C_16_H_12_O_6_	301.0707	301.0702	1.5478	301.07019
26	Naringenin	8.68	C_15_H_12_O_5_	273.0758	273.0753	1.6516	167.0338
27	Tricin methyl ether	12.20	C_18_H_16_O_7_	345.0969	345.0963	1.6836	345.09893
28	Angolensin	4.69	C_16_H_16_O_4_	273.1121	273.1126	1.6989	137.05802
29	Syringetin 3-galactoside	9.30	C_23_H_24_O_13_	509.1290	509.1281	1.7108	347.07443
30	Epigallocatechin	7.34	C_15_H_14_O_7_	307.0812	307.0807	1.7292	307.1301
31	Astragalin	8.88	C_21_H_20_O_11_	449.1078	449.1087	1.9127	287.05789
32	Plantaginin	10.01	C_21_H_20_O_11_	449.1078	449.1087	1.9127	287.0553
33	Gallocatechin	8.70	C_15_H_14_O_7_	307.0812	307.0806	2.0548	163.03957
34	Kakkalide	11.43	C_28_H_32_O_15_	609.1814	609.1827	2.1324	315.08939
35	Icariside II	8.96	C_27_H_30_O_10_	515.1912	515.1923	2.1817	177.05557
36	Velutin	18.27	C_17_H_14_O_6_	315.0863	315.0871	2.4882	315.0876
37	Tectorigenin	13.01	C_16_H_12_O_6_	301.0707	301.0699	2.5443	301.07271
38	Neodiosmin	12.73	C_28_H_32_O_15_	609.1814	609.1830	2.6248	361.09403
39	Demethoxycentaureidin 7-O-rutinoside	10.50	C_29_H_34_O_16_	639.1920	639.1902	2.7629	315.08934
40	Kushenol I	18.06	C_26_H_30_O_7_	455.2064	455.2051	2.9239	455.20299
41	Isorhamnetin 3-O-neohesperoside	5.43	C_28_H_32_O_16_	625.1763	625.1744	3.0647	625.17942
42	Andrographidin B	10.56	C_23_H_24_O_12_	493.1341	493.1356	3.1310	331.08253
43	Rhamnazin	10.71	C_17_H_14_O_7_	331.0812	331.0823	3.2288	331.08269
44	6”-O-Malonylglycitin	14.51	C_25_H_24_O_13_	533.1290	533.1307	3.2431	345.09427
45	Sutchuenoside A	14.64	C_29_H_32_O_15_	621.1814	621.1793	3.3823	315.08714
46	Oenin	15.57	C_23_H_24_O_12_	493.1341	493.1358	3.5366	493.16754
47	Camellianin B	6.24	C_27_H_30_O_14_	579.1708	579.1729	3.5637	144.0809
48	Germanaism E	13.40	C_29_H_32_O_16_	637.1763	637.1786	3.5846	144.0809
49	6-Prenylnaringenin	7.57	C_20_H_20_O_5_	341.1384	341.1397	3.9544	137.05797
50	eupomatenoid 5	5.73	C_19_H_18_O_3_	295.1329	295.1317	3.9677	120.08065
51	Jacein	11.41	C_24_H_26_O_13_	523.1446	523.1467	3.9740	361.09361
52	Iridin	8.20	C_24_H_26_O_13_	523.1446	523.1467	3.9740	523.29549
53	Hexandraside F	11.19	C_39_H_50_O_20_	839.2968	839.3002	4.0200	345.099
54	Gossypetin 3,3′,8-trimethylether	11.30	C_18_H_16_O_8_	361.0918	361.0903	4.1430	331.04442
55	Isorhamnetin	9.93	C_16_H_12_O_7_	317.0656	317.0669	4.1600	317.06484
56	Mirificin	14.60	C_26_H_28_O_13_	549.1603	549.1629	4.7873	345.09951
57	Farrerol	8.86	C_17_H_16_O_5_	301.1071	301.1085	4.8122	167.05907
58	Sophoricoside	10.03	C_21_H_20_O_10_	433.1129	433.1108	4.9087	271.05994
59	Eriodictyol	9.39	C_15_H_12_O_6_	289.0707	289.0721	4.9607	163.04137
60	3′,4′,6-Trihydroxy-3,5,7-trimethoxyflavone	14.99	C_18_H_16_O_8_	361.0918	361.0936	4.9960	361.09153

**Figure 4:**
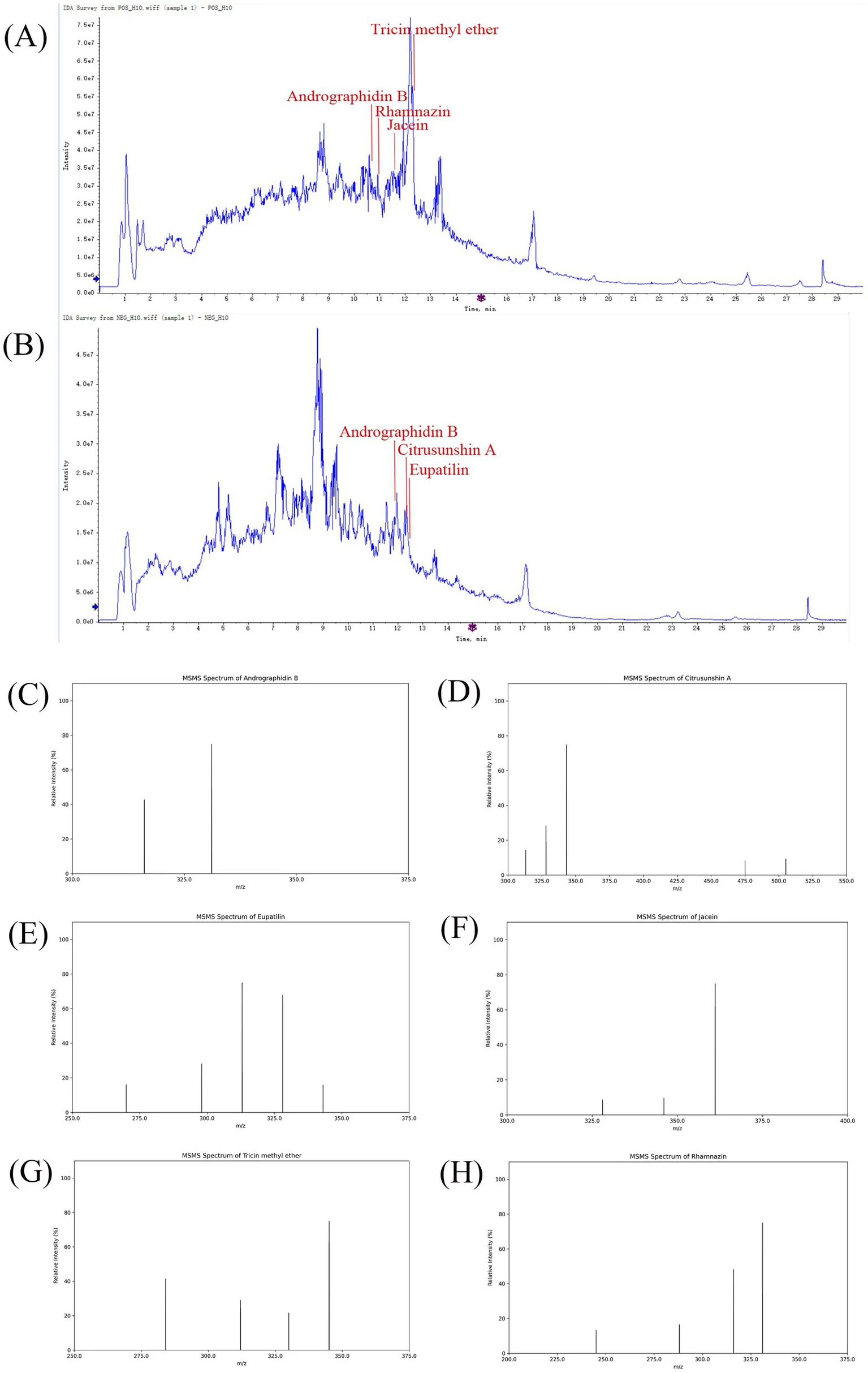
Representative total ion chromatograms (TICs) and MS/MS spectra of key components in TFC. **(A)** TIC acquired in positive ion mode; **(B)** TIC acquired in negative ion mode; **(C)** Andrographidin B; **(D)** Citrusunshin A; **(E)** Eupatilin; **(F)** Jacein; **(G)** Tricin methyl ether; **(H)** Rhamnazin. Labels adjacent to chromatographic peaks in panels **(A,B)** denote approximate peak heights corresponding to the experimental retention times of these six flavonoids.

### 3.3 Antimicrobial activity

#### 3.3.1 Inhibitory zone diameter of TFC

As shown in Figure 5, the negative control (DMSO) did not produce an inhibition zone, confirming that the solvent itself had no antibacterial effect and did not interfere with the assay. In contrast, TFC exhibited significant antibacterial activity against the tested strains in a concentration-dependent manner. The diameters of the inhibition zones and the corresponding susceptibility interpretations are summarized in Table 8.

**Figure 5:**
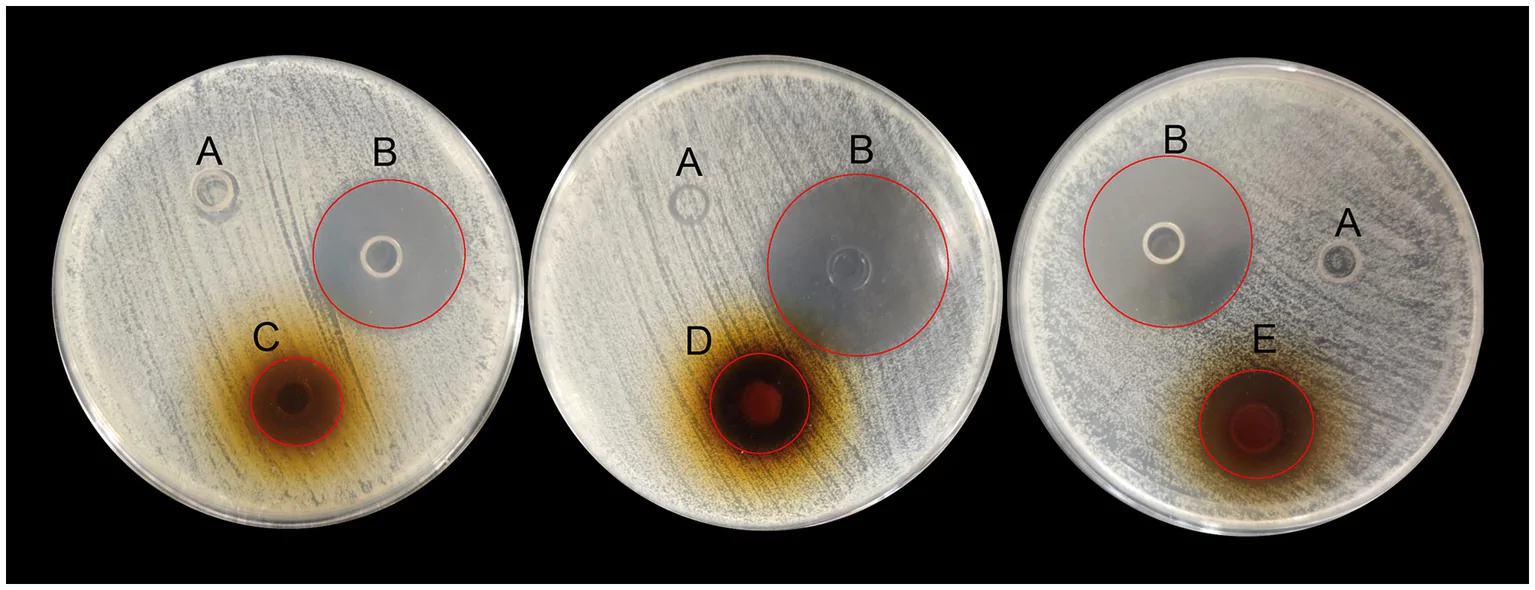
Inhibitory zone of TFC against *S. aureus*. (A) DMSO; (B) Erythromycin (100 μg/mL); (C) TFC (50 mg/mL); (D) TFC (100 mg/mL); (E) TFC (150 mg/mL). *n* = 3 in each group.

**Table 8:** Inhibition zone diameters and susceptibility interpretation of TFC against the *S. aureus*.

Treatment	Inhibitory zone diameter(mm)	Interpretation
DMSO	0 ± 0	No inhibition
Erythromycin(100 μg/mL)	30 ± 0.41	Highly sensitivity
TFC (50 mg/mL)	13 ± 0.45	Moderately sensitivity
TFC (100 mg/mL)	16 ± 0.38	Moderately sensitivity
TFC (150 mg/mL)	20 ± 0.45	Highly sensitivity

An inhibitory zone diameter less than 7 mm indicated no inhibitory effect; *n* = 3 in each group. A diameter between 7 and 10 mm indicated low sensitivity; a diameter greater than 10 mm but less than 20 mm indicated moderate sensitivity; a diameter greater than 20 mm indicated high sensitivity.

#### 3.3.2 MIC and MBC of TFC

##### 3.3.2.1 MIC

The MIC of TFC was determined via the broth microdilution method. As shown in Figure 6A, a gradual increase in broth turbidity was observed as the TFC concentration was serially reduced from 25 mg/mL to 0.097 mg/mL. At a TFC concentration of 0.781 mg/mL, the culture broth remained transparent, indicating no observable bacterial growth. In contrast, marked turbidity was observed at 0.390 mg/mL, signifying substantial bacterial proliferation. The positive control (erythromycin) yielded clear wells, whereas the negative control (DMSO) exhibited moderate turbidity, verifying that the solvent exerted no inhibitory interference. Accordingly, the MIC of TFC against *S. aureus* was determined to be 0.781 mg/mL.

**Figure 6:**
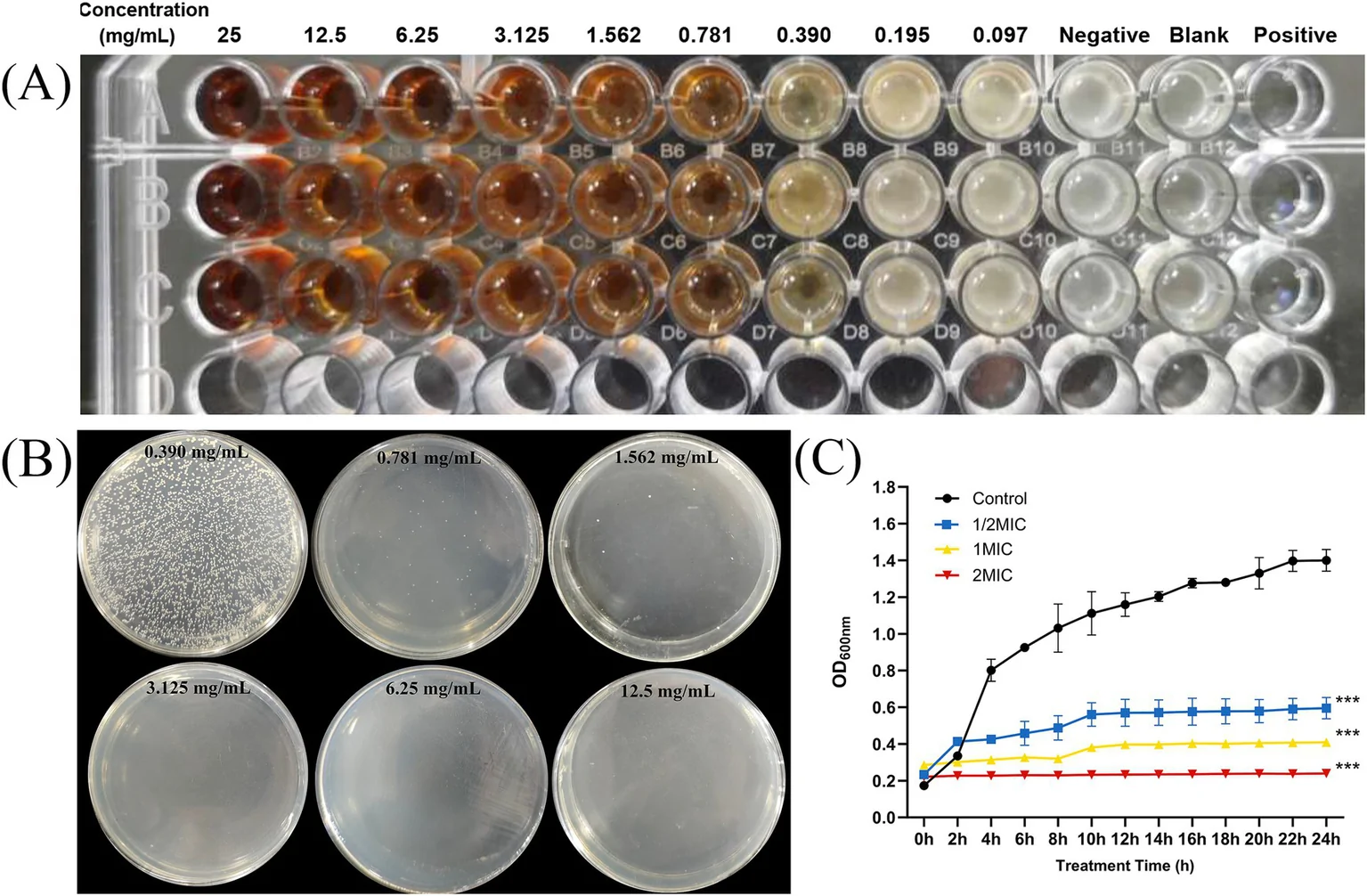
**(A)** MIC determination of TFC against *S. aureus* via broth microdilution method. The wells contained: Negative group: 100 μL *S. aureus* suspension + 100 μL DMSO; Positive group: 100 μL *S. aureus* suspension + 100 μL erythromycin solution; Blank: 200 μL LB broth; TFC group: 100 μL of TFC solution at various concentrations + *S. aureus* suspension. **(B)** Determination of MBC of TFC against *S. aureus*. **(C)** Growth curves of *S. aureus* treated with different concentrations of TFC. Data are expressed as the mean values ± SD, and *n* = 3 in each group; ***p < 0.001, vs. the control.

##### 3.3.2.2 MBC

Based on the MIC results, liquid samples were collected from the clear well (indicating no visible growth) and the well corresponding to 1/2 × MIC, and then spread onto agar plates. When the TFC concentration reached 1.562 mg/mL, the colony count on the agar plates was reduced by 99.9%; therefore, the MBC was determined to be 1.562 mg/mL (Figure 6B). This MBC value is twice the MIC (0.781 mg/mL) and is less than four times the MIC, indicating that TFC exhibits bactericidal activity against *S. aureus* concentrations of 1.562 mg/mL and above.

#### 3.3.3 Bactericidal curve of TFC against *S. aureus*

As shown in Figure 6C, *S. aureus* in the control group entered the exponential growth phase after 2 h, with a continuous increase in OD_600nm_ values. The *S. aureus* treated with 1/2 × MIC showed growth, but at a significantly slower rate. The growth curve of the 1 × MIC group remained nearly flat, indicating a pronounced bacteriostatic effect. In contrast, the 2 × MIC group exhibited no significant variation in OD_600nm_ values, demonstrating complete inhibition of bacterial proliferation at this concentration.

### 3.4 Antibacterial mechanism

#### 3.4.1 CSH of *S. aureus*

CSH is a key indicator reflecting the structural integrity and surface composition of bacterial cell membranes. In this study, the n-hexane adhesion assay was used to measure CSH, as illustrated in Figure 7. After treatment with TFC, *S. aureus* CSH increased significantly (*p* < 0.001) in a concentration-dependent manner. Compared with the control group (18.5%), CSH rose to approximately 32, 54, and 74% in the 0.5 × MIC, 1 × MIC, and 2 × MIC treatment groups, respectively. These results indicate that TFC markedly alters the CSH of *S. aureus*.

**Figure 7:**
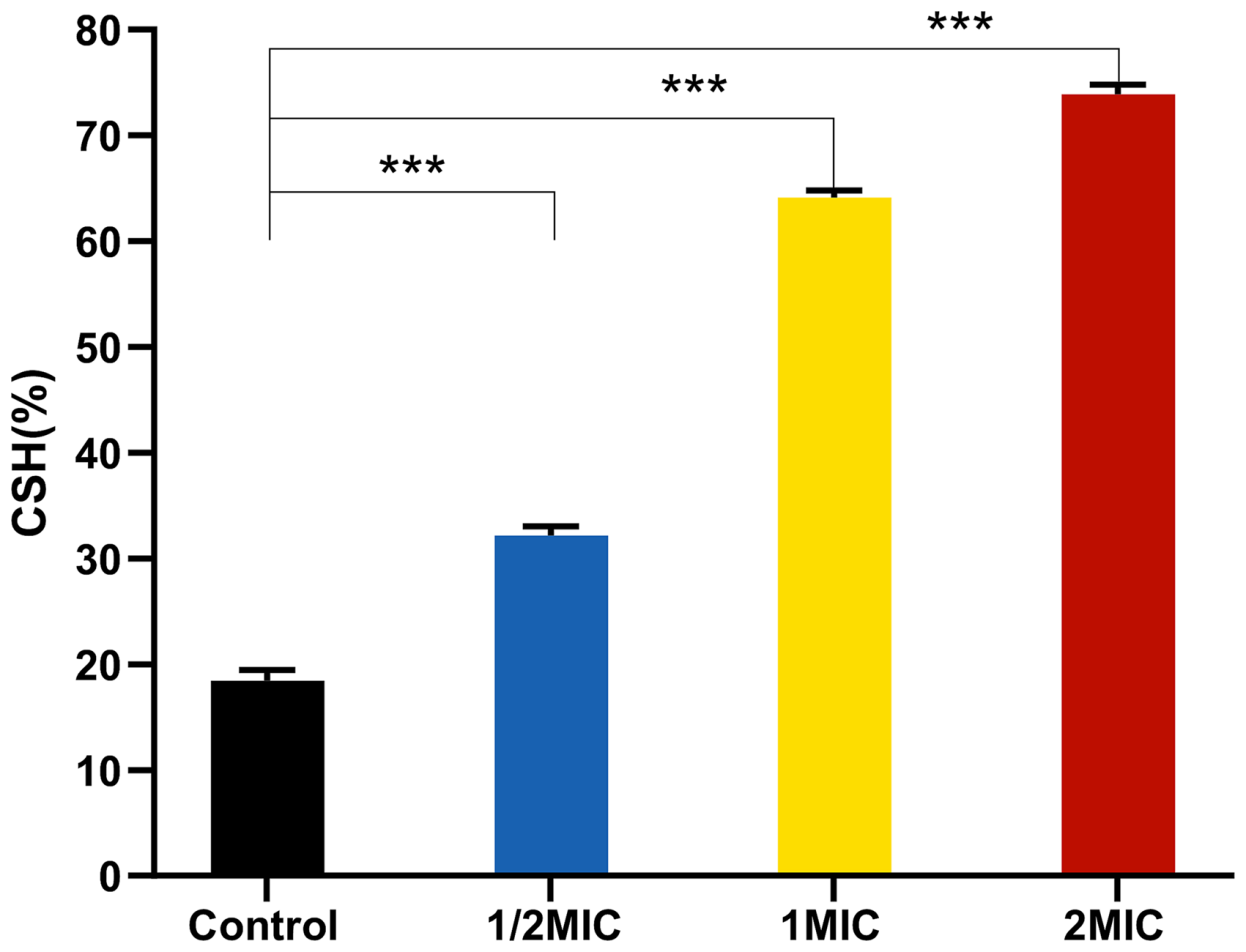
Effect of TFC on the CSH of *S. aureus*. Data are expressed as the mean values ± SD, and *n* = 3 in each group; ***p < 0.001, vs. the control.

#### 3.4.2 Extracellular AKP activity of *S. aureus*

To investigate the effect of TFC on the *S. aureus* cell wall, AKP activity in the supernatant was measured. As shown in Figure 8, after TFC treatment, the OD₄₀₅ values of the supernatant in all treatment groups were significantly higher than that of the control group (*P* < 0.001), and the increase exhibited a concentration-dependent pattern. These results indicate that TFC disrupts the structural integrity of the *S. aureus* cell wall, causing AKP leak into the extracellular environment.

**Figure 8:**
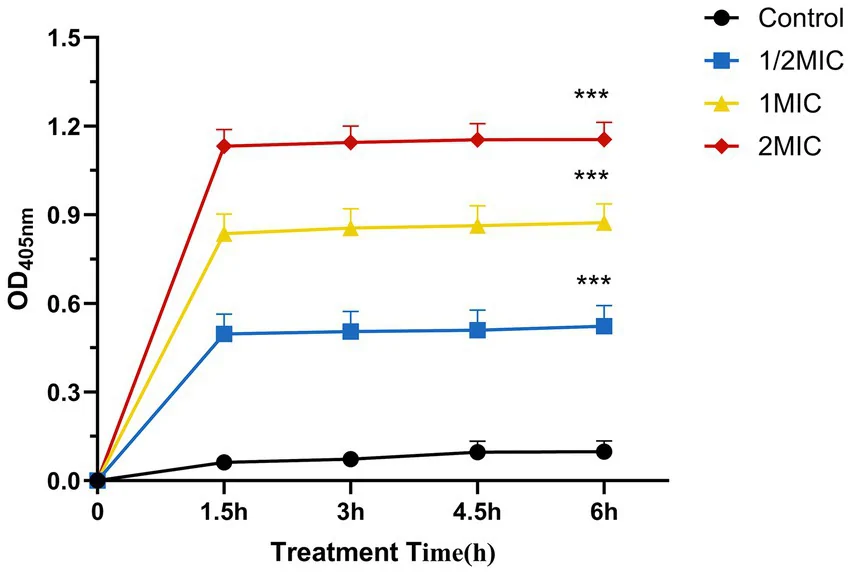
Effect of TFC on extracellular AKP activity of *S. aureus*. Data are expressed as the mean values ± SD, and *n* = 3 in each group; ***p < 0.001, vs. the control.

#### 3.4.3 Extracellular nucleic acid and protein concentrations of *S. aureus*

To evaluate the membrane-damaging effect of TFC, the leakage of intracellular nucleic acids and proteins from *S. aureus* cells was measured. As shown in Figure 9, in the control group, OD₂₆₀ and OD₂₈₀ values remained at low levels, while in TFC treatment group, the OD₂₆₀ and OD₂₈₀ values increased in a TFC concentration-dependent manner, indicating that TFC treatment induced the leakage of intracellular nucleic acids and proteins of *S. aureus*. These data demonstrated that TFC disrupts the integrity of *S. aureus* cell membrane, leading to the efflux of cytoplasmic nucleic acids and proteins.

**Figure 9:**
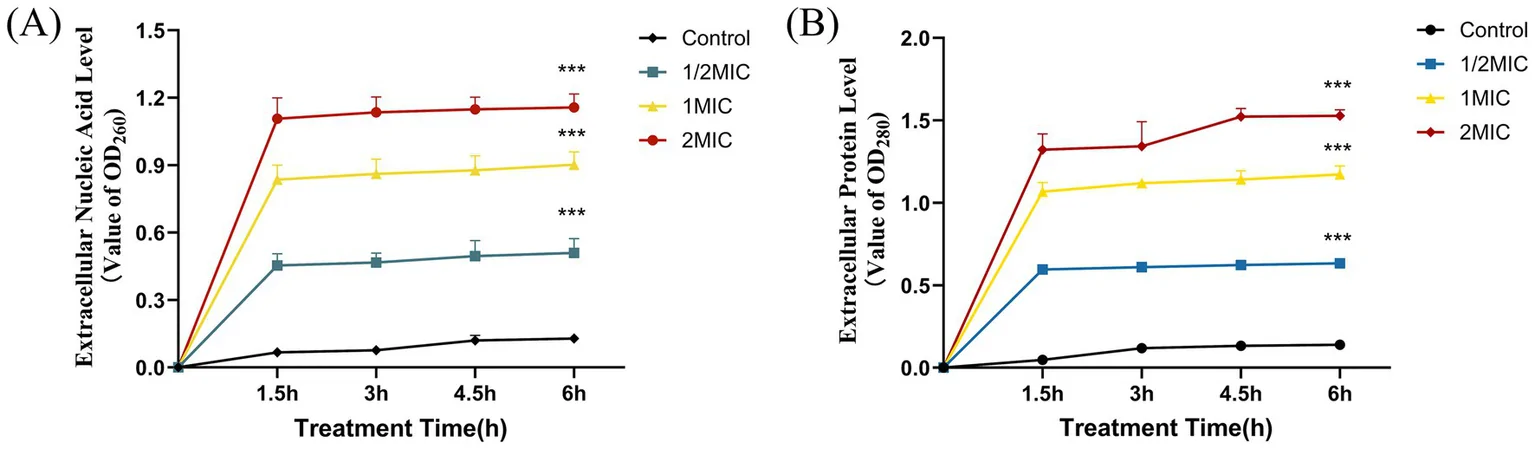
Extracellular nucleic acid and protein levels in *S. aureus* after TFC treatment. **(A)** Extracellular nucleic acid levels; **(B)** Extracellular protein levels. Data are expressed as the mean values ± SD, and *n* = 3 in each group; ***p < 0.001, vs. the control.

#### 3.4.4 Cell membrane potential of *S. aureus*

Cell membrane potential is essential for maintaining bacterial energy metabolism. In this study, the membrane potential-sensitive fluorescent probe Rhodamine 123 was used to evaluate the effect of TFC on the membrane potential of *S. aureus*. As shown in Figure 10, the control group maintained a high negative membrane potential, resulting in intracellular dye accumulation and strong fluorescence signals. In contrast, after treatment with TFC at different concentrations, the fluorescence intensity of *S. aureus* decreased significantly in a concentration-dependent manner (*P* < 0.001). Notably, the fluorescence intensity in the 2 × MIC-treated group was reduced to only about 10% of that in the control. This marked decrease in fluorescence indicates substantial depolarization of the *S. aureus* cell membrane. These results suggest that TFC may disrupt *S. aureus* cellular energy metabolism and bacterial growth by damaging membrane structure, interfering with transmembrane ion homeostasis, and impairing energy production.

**Figure 10:**
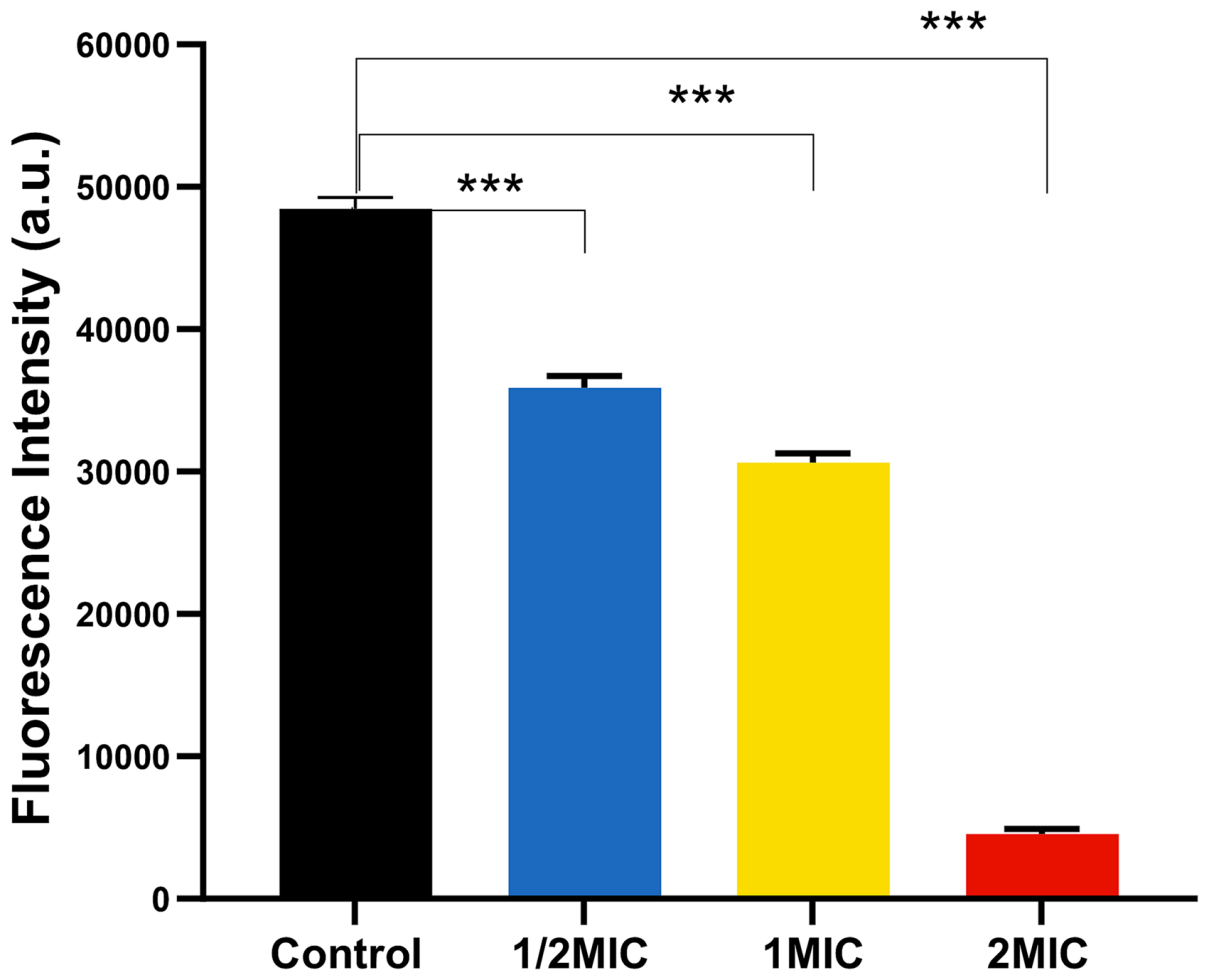
Relative fluorescence intensity of cell membrane potential in *S. aureus*. Data are expressed as the mean values ± SD, and *n* = 3 in each group; ***p < 0.001, vs. the control.

#### 3.4.5 Intracellular nucleic acids of *S. aureus*

To investigate the effect of TFC on the structural integrity of nucleic acids, the fluorescence intensity of DNA and RNA in *S. aureus* were measured using DAPI staining. As shown in Figure 11, compared with the control group, TFC treatment resulted in a highly a significant decrease in the fluorescence intensity of both DNA and RNA (*p* < 0.001). Moreover, as the TFC concentration increased from 1/2 × MIC to 2 × MIC, the fluorescence intensities of DNA and RNA decreased in a concentration-dependent manner. These findings indicated that TFC reduces the intracellular nucleic acid content in *S. aureus* and/or causes structural damage to nucleic acids, suggesting that its antibacterial activity may involve the disruption of DNA/RNA structures or the promotion of nucleic acid degradation.

**Figure 11:**
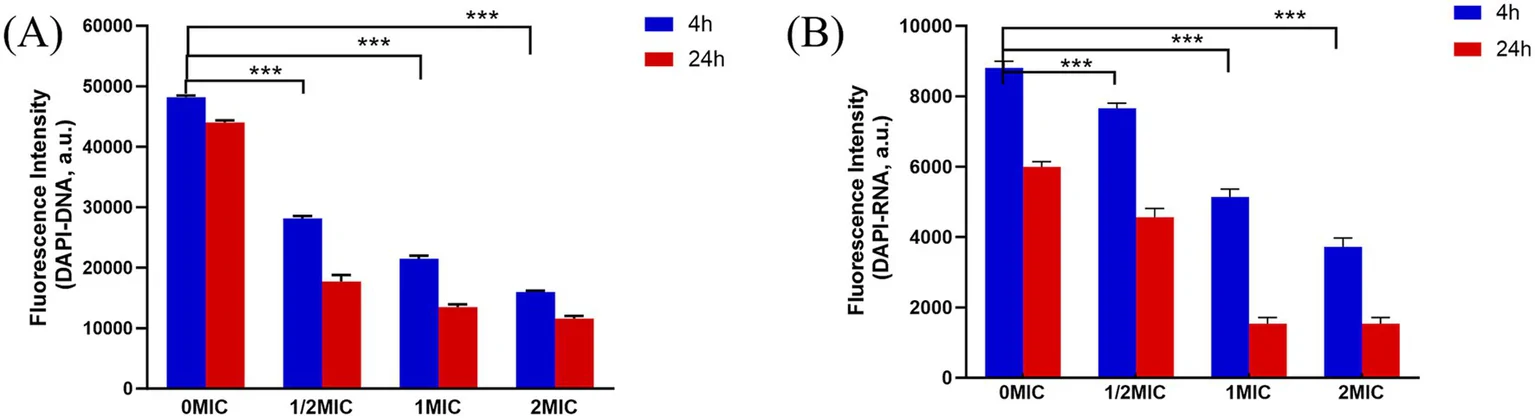
Effect of TFC on the fluorescence intensity of DNA in *S. aureus*. **(A)** Fluorescence intensity of DNA; **(B)** Fluorescence intensity of RNA. Data are expressed as the mean values ± SD, and *n* = 3 in each group; ***p < 0.001, vs. the control.

#### 3.4.6 Intracellular protein of *S. aureus*

The expression profile of total bacterial proteins was analyzed by SDS-PAGE, and the results are shown in Figure 12. The lane labelling system used in the gel follows the format “Time_Treatment_.” The numeric prefix denotes the sampling time point in hours (4, 16, or 24). The subscript denotes the treatment condition: “0” for the untreated control, “1/2” for 1/2 × MIC, “1” for 1 × MIC, and “2” for 2 × MIC. For instance, lane 4_0_ represents the control group at 4 h, whereas lane 16_2_ represents the 2 × MIC treatment group at 16 h. The blank control group (lanes ending with “0,” e.g., 4_0_, 16_0_, 24_0_) displayed abundant protein bands across a wide molecular weight range at 4 h, 16 h, and 24 h. Moreover, the band intensity increased over time, indicating a rise in protein content and confirming intact protein synthesis and metabolic activity under untreated conditions. In the 1/2 × MIC treatment group (lanes ending with “1/2”), visible attenuation was observed in some protein bands, suggesting that the synthesis of certain specific proteins was affected at an early stage. When the concentration was increased to 1 × MIC (lanes ending with “1”), the signal intensity of most medium- to high-abundance protein bands was significantly reduced. Particularly noteworthy, under the 2 × MIC high concentration treatment (lanes ending with “2”), almost no clearly defined protein bands with sufficient signal intensity were detectable on the gel, with only faint band traces remaining in a very limited molecular weight region (30–42 kDa). These results demonstrate that TFC may inhibit the growth and metabolism of *S. aureus* by suppressing protein synthesis and/or promoting protein degradation, thereby reducing the total protein content.

**Figure 12:**
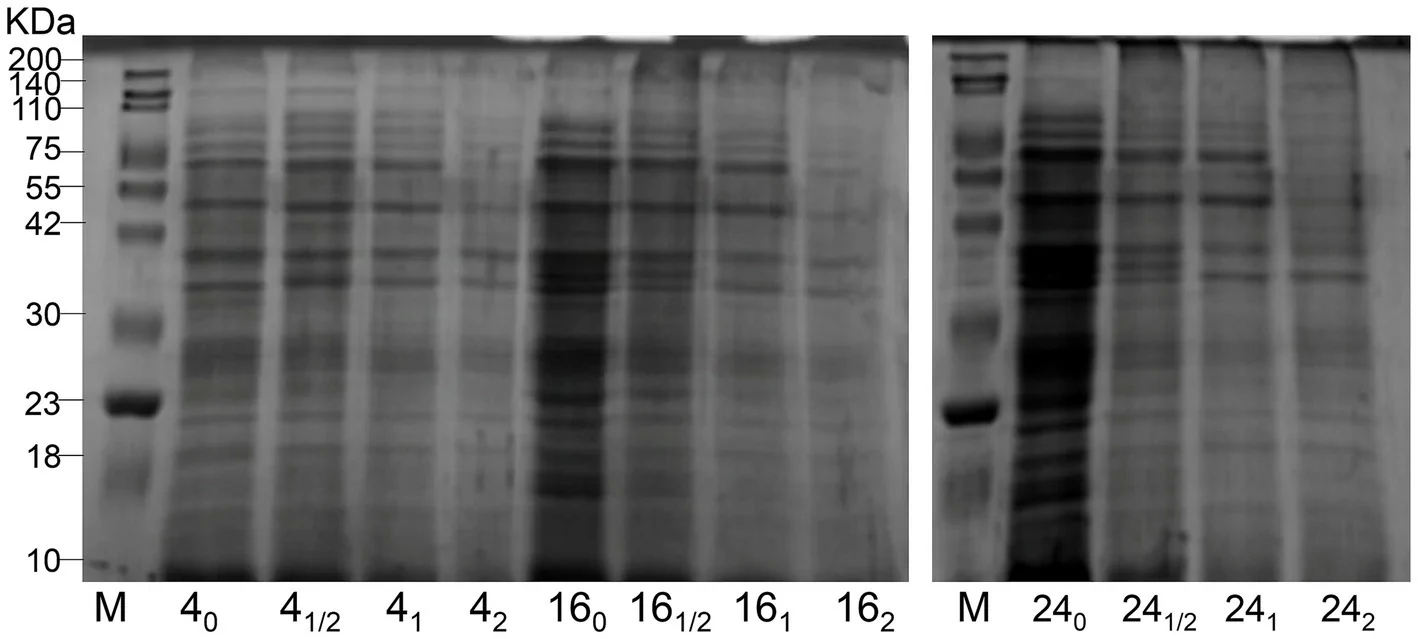
SDS-PAGE analysis of total protein expression in *S. aureus* treated with TFC. Lane M: Protein molecular weight marker. The numeric prefix indicates the sampling time point in hours (4, 16, or 24). The subscript indicates the treatment condition: 0 (untreated control), 1/2 (1/2 × MIC), 1 (1 × MIC), and 2 (2 × MIC). Thus, lanes such as 4_0_, 4_1/2_, 4_1_, and 4_2_ represent samples collected at 4 h under control, 1/2 × MIC, 1 × MIC, and 2 × MIC treatments, respectively; similarly for 16 h (16_0_–16_2_) and 24 h (24_0_–24_2_).

#### 3.4.7 ROS levels in *S. aureus*

To investigate the effect of TFC on oxidative stress in *S. aureus*, the intracellular ROS levels were measured using the DCFH-DA fluorescent probe. As shown in Figure 13, compared to the control group, *S. aureus* treated with TFC exhibited a highly significant increase in ROS fluorescence intensity (*p* < 0.001). Furthermore, the fluorescence intensity increased gradually with prolonged treatment duration and higher TFC concentration. These findings indicated that TFC induces substantial ROS production in *S. aureus*, thereby inducing severe oxidative stress and ultimately contributing to bacterial death.

**Figure 13:**
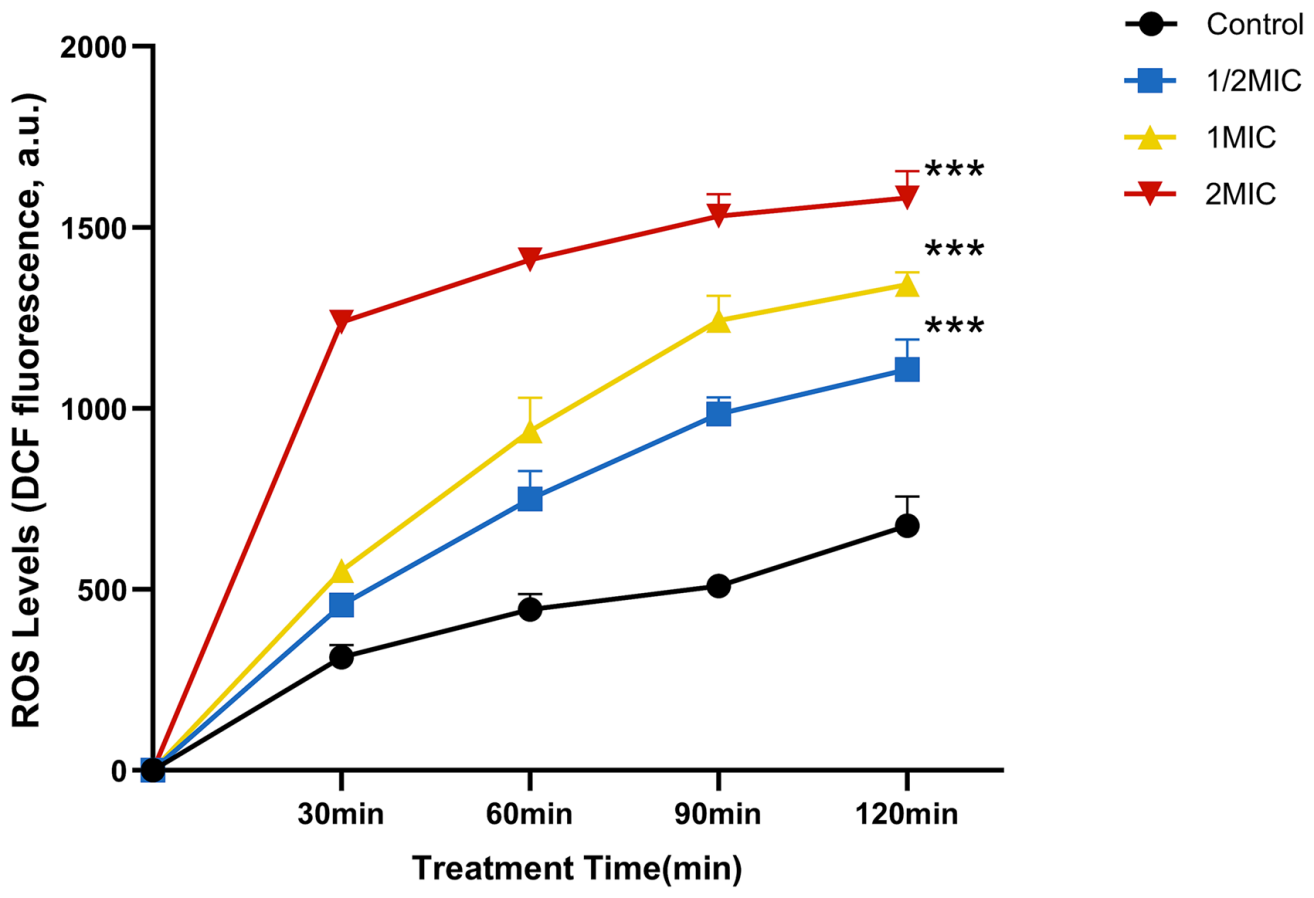
ROS levels in *S. aureus* treated with different concentrations of TFC over time. Data are expressed as the mean values ± SD, and *n* = 3 in each group; ***p < 0.001, vs. the control.

#### 3.4.8 SEM analysis

SEM analysis revealed significant morphological alterations in *S. aureus* following TFC treatment. Untreated control cells exhibited typical grape-like clustering, presenting as regular cocci with extremely smooth, turgid surfaces, the cells were uniform in size, clearly demarcated, and free from any signs of adhesion, indentation, or structural damage (Figures 14A,D). In contrast, TFC-exposed bacteria underwent severe degenerative changes and irreversible structural damage. In the 1 × MIC treatment group, the characteristic smoothness of the cell surface was disrupted, giving way to distinct granular roughness. Partial cells exhibited mild shrinkage with reduced volume and formed irregular aggregates with adjacent cells (Figures 14B,E). In the 2 × MIC treatment group, a substantial proportion of the population displayed severe collapse, distortion and deflation, resulting in a complete loss of the original spherical morphology. Localized sites of membrane rupture were evident, accompanied by probable leakage of intracellular contents (Figures 14C,F). These observations demonstrate that TFC compromises the structural integrity of the *S. aureus* cell walls and membranes, inducing surface coarsening, cellular shrinkage and eventual lysis, thereby leading to cytoplasmic leakage and exerting potent bactericidal effects.

**Figure 14:**
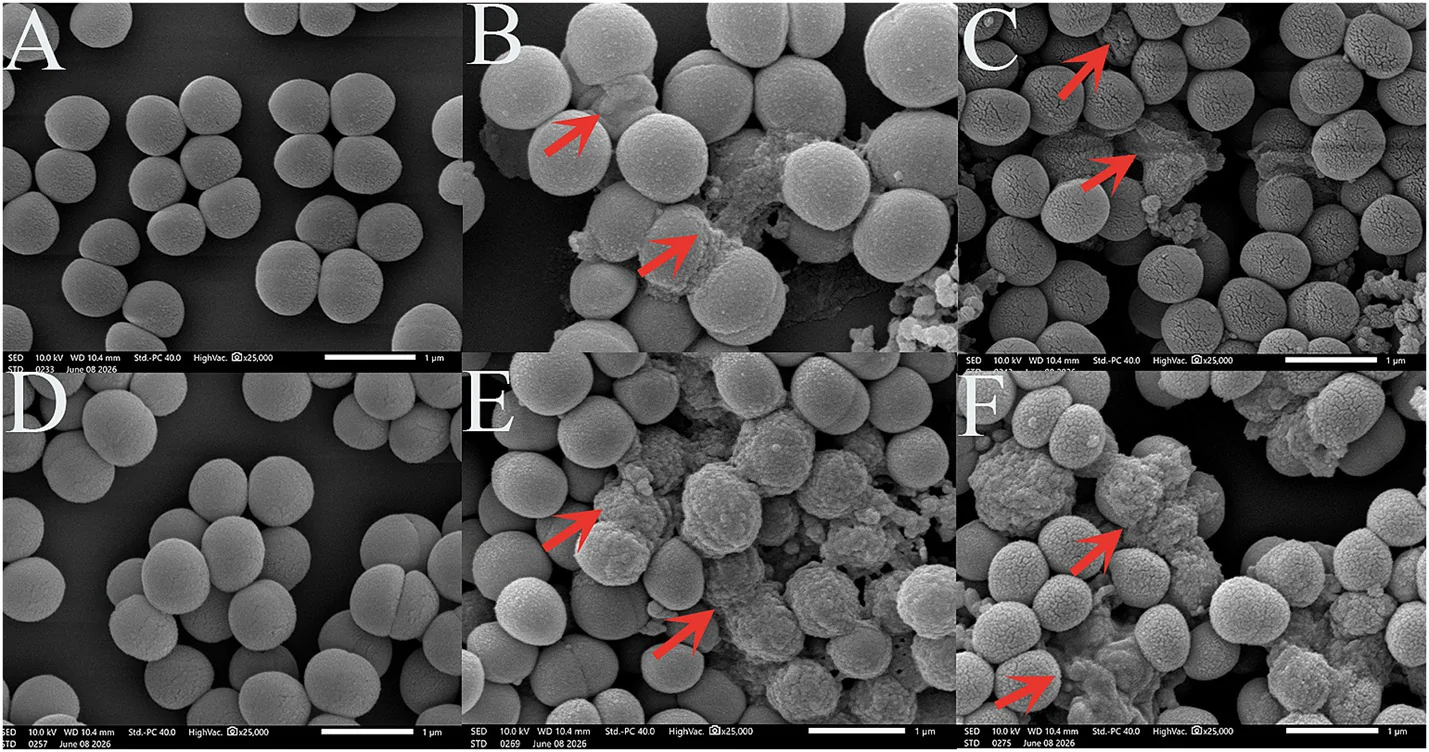
Scanning electron micrographs of *S. aureus*. Untreated controls at 6 h **(A)** and 12 h **(D)** post-inoculation, respectively. Cells treated with TFC at 1 × MIC for 6 h **(B)** and 12 h **(E)**, respectively. Cells treated with TFC at 2 × MIC for 6 h **(C)** and 12 h **(F)**, respectively. The arrows point to the morphological changes in the cells. Scale bars, 1 μm; height, ×25,000.

#### 3.4.9 Effect of TFC on intracellular ATP concentration

As shown in Figure 15, TFC treatment reduced intracellular ATP content in *S. aureus* cell in a concentration-dependent manner. Treatment with 1/2 × MIC resulted in a modest ATP reduction, whereas treatment with 1 × MIC and 2 × MIC caused progressively severe ATP depletion. The significant intracellular ATP loss at 2 × MIC indicates severe impairment of *S. aureus* cell integrity and energy metabolism. Collectively, these alterations represent of a key mechanistic basis for the bactericidal activity of TFC against of *S. aureus*.

**Figure 15:**
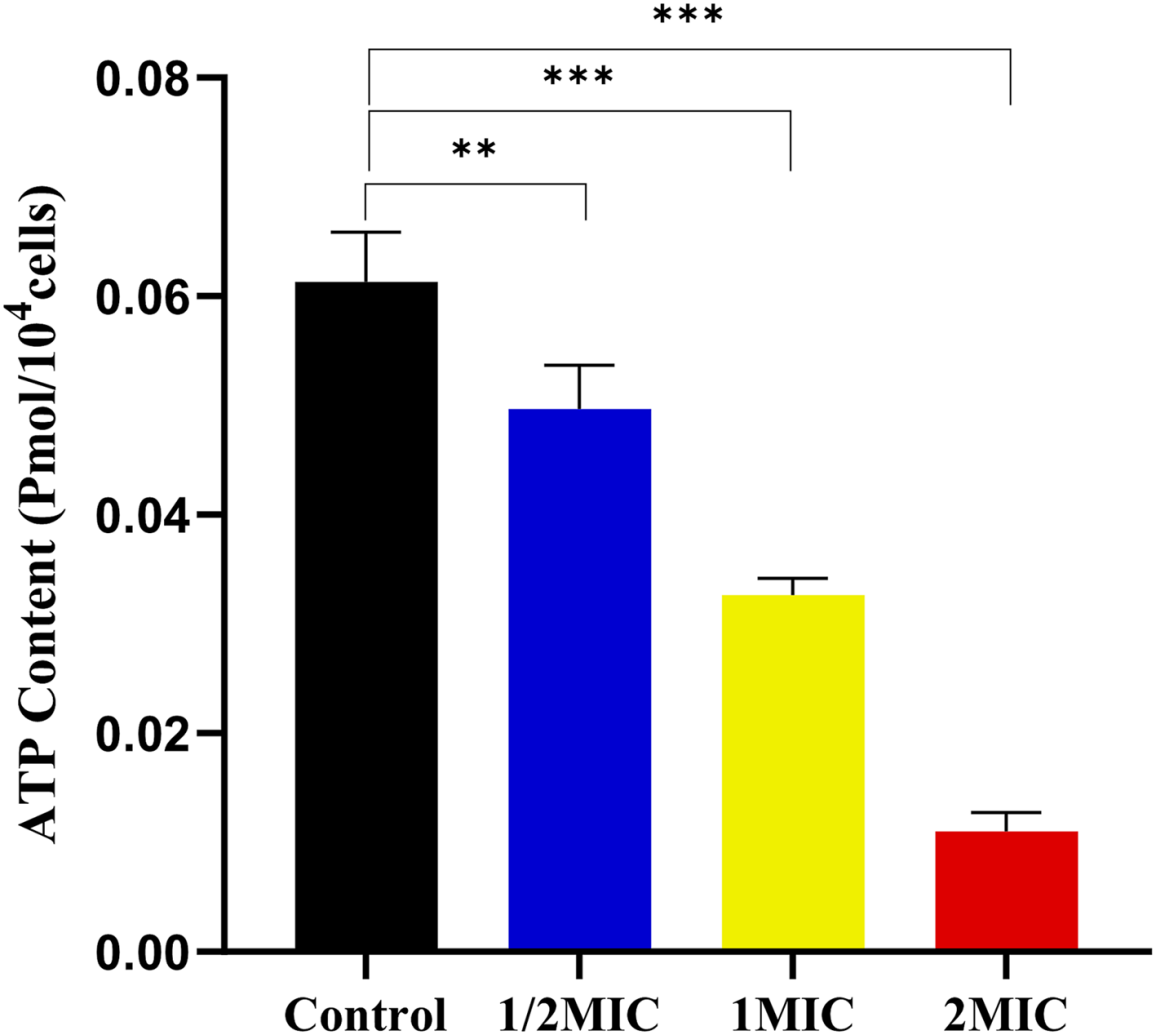
Effects of TFC treatment on intracellular ATP content of *S. aureus*. Data are expressed as the mean values ± SD, and *n* = 3 in each group; **p < 0.01, ***p < 0.001, vs. the control.

## 4 Discussion

*S. aureus*, one of the most prevalent clinical pathogens, presents an increasingly serious therapeutic challenge, largely due to its high virulence and multidrug resistance (Bashabsheh et al., 2024)^.^ As the effectiveness of conventional antibiotics continues to decline, clinical treatment faces the dilemma of “no drugs available” (Wei et al., 2024). TCM compounds hold significant potential to address this challenge owing to their multi-target synergistic antibacterial activity, low propensity to induce resistance, and favorable biosafety profile (Man et al., 2025). Our previous studies have found that the ethanol extract of *Clerodendrum cyrtophyllum* Turcz. exhibits potent antibacterial activity against *S. aureus*, while the antibacterial active components of this plant remain to be further investigated (Pei et al., 2025). Thus, we speculated that flavonoids are the key antibacterial components of *Clerodendrum cyrtophyllum* Turcz. The present study aimed to optimize the extraction conditions for TFC, characterize the TFC profile using LC–MS/MS, and investigate the antibacterial activity and underlying mechanisms of TFC against *S. aureus*.

In terms of extraction process optimization, this study investigated the effects of ethanol concentration, solid-to-liquid ratio, extraction temperature, and extraction time on the TFC yield through single-factor experiments combined with response surface methodology. The optimized extraction condition for TFC is 70% ethanol. Because of an ethanol concentration below 70% resulted in higher solvent polarity, which hindered the dissolution and diffusion of flavonoids. In contrast, a concentration of ethanol above 70% suppressed the release of hydrophilic components and potentially reduced extraction efficiency due to protein denaturation and subsequent adsorption of target compounds (Yusoff et al., 2022). The optimal solid-to-liquid ratio is 1:25 (g: mL). A solid-to-liquid ratio lower than 1:25 (g: mL) provided insufficient solvent for thorough extraction, while a ratio higher than 1:25 excessively diluted the system, weakening the mass transfer driving force (Huang et al., 2020). The optimal extraction temperature was determined to be 64 °C. Lower temperatures (<64 °C) slowed molecular diffusion, whereas higher temperatures (>64 °C) promoted thermal degradation of heat-sensitive flavonoids and increased solvent evaporation (Zhu et al., 2024). The optimal extraction time is 102 min. Shorter durations (<102 min) were insufficient to reach mass transfer equilibrium, while prolonged extraction (>102 min) could lead to thermal degradation or structural transformation of the extracted flavonoids (Ning et al., 2025).

The cell wall is a robust structure surrounding the bacterial cell membrane, and its integrity is essential for bacterial survival and resistance to external stress (Mueller and Levin, 2020). AKP is a marker enzyme mainly localized between the cell wall and membrane. However, when drugs compromise the cell wall, increased membrane permeability allows AKP to leak into the extracellular environment. Therefore AKP is an important indicator for assessing the degree of cell wall damage (Tang et al., 2021). In the present study, a marked increase in AKP activity in the supernatant was observed after TFC treatment, confirming that TFC specifically disrupts the integrity of the *S. aureus* cell wall. This structural damage was further visualized by SEM, which revealed severe morphological alterations, including roughness, collapse, and leakage of intracellular contents from the damaged cell surface. The cell membrane, a key semi-permeable barrier that maintains cellular structure, regulates material transport, participates in energy metabolism and signal transduction, is a major target of antimicrobial agents (Zhukov and Popov, 2023). Once membrane structure is impaired and permeability increases, intracellular nucleic acids and proteins leak into the extracellular medium, leading to a pronounced rise in the absorbance of the supernatant at 260 nm and 280 nm. In the present study, TFC treatment induced substantial leakage of intracellular nucleic acids and proteins, indicating that TFC compromises the integrity of *S. aureus* cell membrane. With respect to membrane function, changes in the cell membrane potential were evaluated using the Rhodamine 123 fluorescent probe. This cationic dye accumulates intracellularly via the electrochemical gradient established by the normal, internally negative membrane potential, yielding a strong fluorescent signal. Membrane depolarization disrupts this gradient, prevents effective dye accumulation, and results in a sharp decrease in fluorescence intensity (Toescu and Verkhratsky, 2000). Our data demonstrate that after TFC treatment, the fluorescence intensity of *S. aureus* decreased significantly in a concentration-dependent manner, indicating that TFC treatment caused depolarization of the bacterial membrane potential. This depolarization is likely to disturb the maintenance of the proton motive force, which in turn may impair energy-dependent processes such as ATP synthesis, ion homeostasis, and transmembrane nutrient transport, ultimately inhibiting *S. aureus* growth. Consistent with the above finding, we observed a significant depletion of intracellular ATP levels following TFC treatment, suggesting that the disruption of membrane potential directly cripples *S. aureus* energy metabolism. Nucleic acids are the genetic material of organisms and the central molecules governing biological traits and life activities. DAPI is a fluorescent dye that binds specifically to nucleic acids. Under specific excitation wavelengths, the DAPI-nucleic acid complex emits characteristic fluorescence, the intensity of which correlates positively with intracellular nucleic acid content and structural integrity (Kapuscinski, 1995). We demonstrated that TFC treatment decreased fluorescence intensity in *S. aureus*, suggesting that it compromises the structural integrity of nucleic acids and/or promotes their degradation. ROS are a group of highly reactive oxygen-containing molecules or free radicals produced during normal cellular metabolism. However, excessive accumulation of ROS can attack lipids, proteins, and DNA, leading to cellular damage (Rosseti et al., 2015). The findings of this study revealed that the intracellular ROS of *S. aureus* was significantly elevated after TFC treatment, indicating that TFC not only directly damages the physical structure of the cell wall and membrane but also by inducing excessive endogenous ROS accumulation, ultimately attacking critical bacterial biomolecules and leading to cell death. Proteins are the executors of bacterial life activities, and the integrity of their structure and function directly determines bacterial viability (Malarney and Chang, 2023). In this study, the expression levels of proteins were assessed by comparing the band intensity in SDS-PAGE gels between treated and control groups, with wider and darker bands indicating higher protein content in the corresponding lane (Wang et al., 2024). Our results demonstrated that TFC exerts its antibacterial activity by interfering with protein biosynthesis and/or inducing protein denaturation, thereby providing a proteomic-level insights into the antibacterial mechanism of TFC against *S. aureus*. From a chemical perspective, comprehensive LC–MS/MS profiling unambiguously annotated 85 distinct flavonoid constituents in the purified TFC fraction, providing a robust phytochemical basis for the observed bioactivity. It is highly plausible that these structurally diverse compounds exert synergistic or additive effects, underscoring the superiority of the complex TFC matrix over isolated single compounds. This chemical complexity could broaden the antibacterial target spectrum, and may play a role in shaping bacterial resistance-related responses.

Unlike most studies that focus on only one or a small number of mechanistic indicators, this study employed a comprehensive panel of assays, including cell-wall integrity assay, cell-membrane permeability evaluation, CSH assay, oxidative stress measurement, energy and protein metabolism studies, and SEM analysis. Collectively, these data confirm that the effect of TFC against *S. aureus* is attributable to the synergistic action of multiple components, implying a more complex mode of action. This aligns with the “multi-component, multi-target” characteristic of traditional Chinese medicine. Although multiple lines of evidence substantiate the apparent *in vitro* anti- *S. aureus* potential of TFC, this study is limited to *in vitro* experiments. Future studies should combine mammalian cell cytotoxicity profiling with suitable infection models, so as to translate the above compositional insights into a rigorous foundation for exploring plant-derived anti- *S. aureus* exploration.

## 5 Conclusion

In this study, the extraction conditions of TFC were optimized to acquire flavonoid-rich extracts. TFC exerts potent inhibitory effects on *S. aureus* via destroying the structural integrity of cell walls and membranes, depleting intracellular energy reserves, decreasing the contents of intracellular nucleic acids and proteins, and triggering oxidative stress responses in bacterial cells.

## Data Availability

The original contributions presented in the study are included in the article/Supplementary material, further inquiries can be directed to the corresponding author.
