# Supplementary material for: Optimization of extraction conditions for total flavonoids from Clerodendrum cyrtophyllum Turcz. and its antibacterial activity and mechanism against Staphylococcus aureus
Source: Front Microbiol. 2026 Sep 21;17:1961127. doi: 10.3389/fmicb.2026.1961127 (PMC13635108; doi:10.3389/fmicb.2026.1961127)
Supplement: Supplementary file 1 [file Data_Sheet_1.PDF]

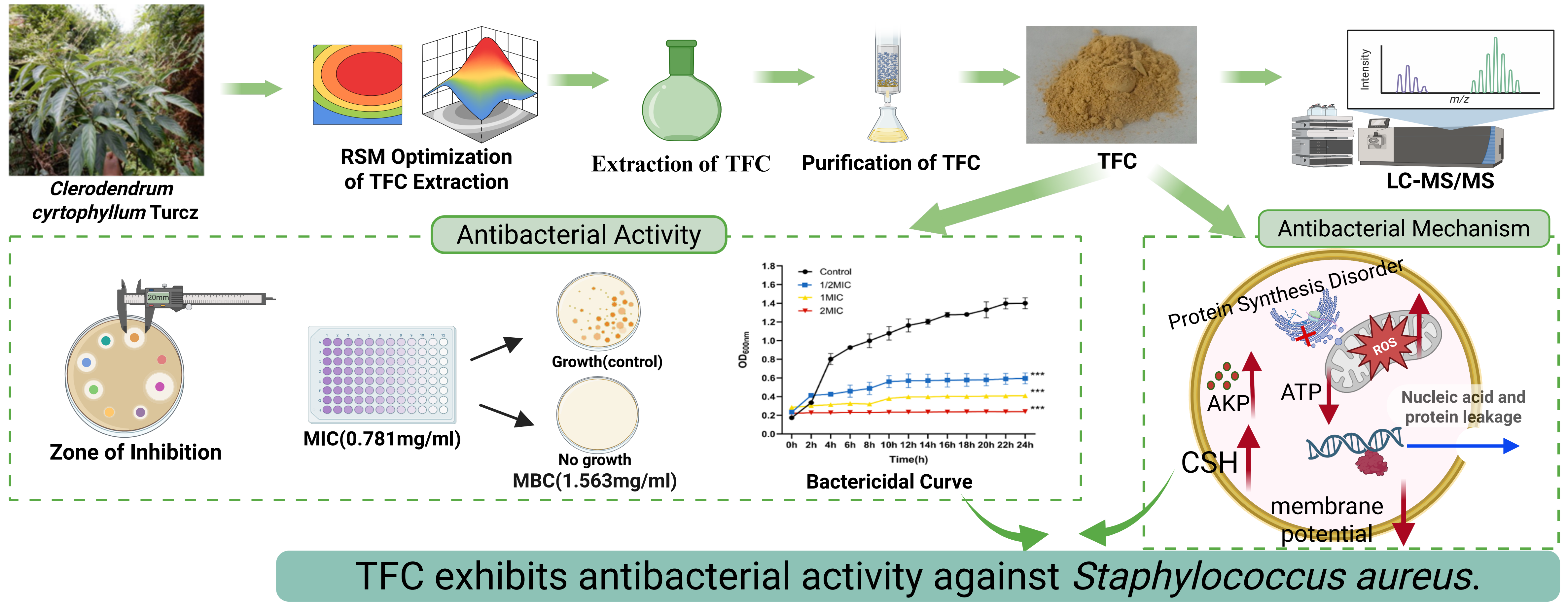

Clerodendrum cyrtophyllum Turcz
RSM Optimization of TFC Extraction
Extraction of TFC
Purification of TFC
TFC
Intensity
m/z
LC-MS/MS
Antibacterial Activity
20mm
Zone of Inhibition
MIC(0.781mg/ml)
Growth(control)
No growth
MBC(1.563mg/ml)
Control
1/2MIC
1MIC
2MIC
OD600nm
Time(h)
Bactericidal Curve
Antibacterial Mechanism
Protein Synthesis Disorder
ROS
AKP
ATP
Nucleic acid and protein leakage
CSH
membrane potential
TFC exhibits antibacterial activity against Staphylococcus aureus.
